# CDC Guidelines for the Prevention and Treatment of Anthrax, 2023

**DOI:** 10.15585/mmwr.rr7206a1

**Published:** 2023-11-17

**Authors:** William A. Bower, Yon Yu, Marissa K. Person, Corinne M. Parker, Jordan L. Kennedy, David Sue, Elisabeth M. Hesse, Rachel Cook, John Bradley, Jürgen B. Bulitta, Adolf W. Karchmer, Robert M. Ward, Shana Godfred Cato, Kevin Chatham Stephens, Katherine A. Hendricks

**Affiliations:** ^1^Division of High-Consequence Pathogens and Pathology, CDC, Atlanta, Georgia; ^2^Division of Preparedness and Emerging Infections, CDC, Atlanta, Georgia; ^3^University of California San Diego School of Medicine, San Diego, California; ^4^University of Florida College of Pharmacy, Orlando, Florida; ^5^Beth Israel Deaconess Medical Center, Boston, Massachusetts; ^6^University of Utah School of Medicine, Salt Lake City, Utah; ^7^Division of Birth Defects and Infant Disorders, CDC, Atlanta, Georgia; ^8^Division of Human Development and Disability, CDC, Atlanta, Georgia

## Abstract

**This report updates previous CDC guidelines and recommendations on preferred prevention and treatment regimens regarding naturally occurring anthrax. Also provided are a wide range of alternative regimens to first-line antimicrobial drugs for use if patients have contraindications or intolerances or after a wide-area aerosol release of:**

Bacillus anthracis* spores if resources become limited or a multidrug-resistant *B. anthracis* strain is used *(Hendricks KA, Wright ME, Shadomy SV, et al.; Workgroup on Anthrax Clinical Guidelines. Centers for Disease Control and Prevention expert panel meetings on prevention and treatment of anthrax in adults. Emerg Infect Dis 2014;20:e130687; Meaney-Delman D, Rasmussen SA, Beigi RH, et al. Prophylaxis and treatment of anthrax in pregnant women. Obstet Gynecol 2013;122:885−900; Bradley JS, Peacock G, Krug SE, et al. Pediatric anthrax clinical management. Pediatrics 2014;133:e1411-36)*. Specifically, this report updates antimicrobial drug and antitoxin use for both postexposure prophylaxis (PEP) and treatment from these previous guidelines best practices and is based on systematic reviews of the literature regarding 1) in vitro antimicrobial drug activity against *B. anthracis*; 2) in vivo antimicrobial drug efficacy for PEP and treatment; 3) in vivo and human antitoxin efficacy for PEP, treatment, or both; and 4) human survival after antimicrobial drug PEP and treatment of localized anthrax, systemic anthrax, and anthrax meningitis.*

**Changes from previous CDC guidelines and recommendations include an expanded list of alternative antimicrobial drugs to use when first-line antimicrobial drugs are contraindicated or not tolerated or after a bioterrorism event when first-line antimicrobial drugs are depleted or ineffective against a genetically engineered resistant:**

B. anthracis* strain. In addition, these updated guidelines include new recommendations regarding special considerations for the diagnosis and treatment of anthrax meningitis, including comorbid, social, and clinical predictors of anthrax meningitis. The previously published CDC guidelines and recommendations described potentially beneficial critical care measures and clinical assessment tools and procedures for persons with anthrax, which have not changed and are not addressed in this update. In addition, no changes were made to the Advisory Committee on Immunization Practices recommendations for use of anthrax vaccine *(Bower WA, Schiffer J, Atmar RL, et al. Use of anthrax vaccine in the United States: recommendations of the Advisory Committee on Immunization Practices, 2019. MMWR Recomm Rep 2019;68[No. RR-4]:1–14)*. The updated guidelines in this report can be used by health care providers to prevent and treat anthrax and guide emergency preparedness officials and planners as they develop and update plans for a wide-area aerosol release of *B. anthracis*.*

## Introduction

Anthrax is a zoonotic disease caused by infection with *Bacillus anthracis* and still occurs in agricultural regions of the Americas, sub-Saharan Africa, central and southwestern Asia, and southern and eastern Europe (*1*). Sheep, goats, cattle, and other herbivores are primarily affected. Humans are secondarily infected through contact with infected animals, contaminated animal products (e.g., meat or hides), or, rarely, from injection drug use (*2*). Anthrax in humans usually is characterized by the route of *B. anthracis* inoculation (*3*). Cutaneous anthrax, which results from direct inoculation of spores through the skin, is the most common form and accounts for >95% of human cases. Ingestion anthrax usually results from consumption of infected meat. Inhalation anthrax results from the inhalation of aerosolized spores. Injection anthrax, which is a relatively new form, results from injection of heroin contaminated with *B. anthracis* spores. Anthrax meningitis can complicate any form of anthrax or occur alone.

In the United States, anthrax has almost been eliminated through livestock vaccination. Wildlife and livestock anthrax still occurs sporadically in an area from southwest Texas through Colorado, North and South Dakota, and Montana (*4*,*5*). Since 2006, nine confirmed or probable U.S. cases of anthrax have been reported to CDC: two inhalation (*6*,*7*), one ingestion (*8*), four cutaneous (*9*,*10*), and two with no documented route of infection. In addition, since 1997, seven cases of severe pneumonia have been identified that were caused by *Bacillus cereus* group species that harbor a plasmid that encodes anthrax toxins similar to those found in *B. anthracis* (*11*).

*B. anthracis* is a Tier 1 select agent and considered one of the most likely bioterrorism agents to be used because it is relatively easy to acquire from the natural environment, mass produce, and disseminate as spores via aerosolization (*12*). Although approximately 180 countries have signed on to the 1975 Biological Weapons Convention prohibiting the development, production, acquisition, transfer, stockpiling, and use of bioweapons, a wide-area aerosol release of *B. anthracis* spores remains a concern. In 2001, *B. anthracis* spores were sent in letters through the U.S. Postal Service. Exposure to aerosolized spores in these letters resulted in 11 inhalation and 11 cutaneous cases; five inhalation cases were fatal (*13*,*14*). A wide-area aerosol release of *B. anthracis* spores would likely result in a mass-casualty incident (*15*,*16*) that could possibly be complicated by use of genetically engineered *B. anthracis* strains resistant to first-line antimicrobial drugs for postexposure prophylaxis (PEP) and treatment. In 2015, CDC published recommendations for hospital-based acute care that addressed antitoxin and intravenous (IV) antimicrobial drug use and the diagnosis and management of common anthrax-specific complications during a mass-casualty incident that would require a shift from conventional to contingency or crisis standards of care (*17*).

*B. anthracis* possesses three primary virulence factors: an extracellular capsule and two bipartite exotoxins (lethal toxin [composed of lethal factor and protective antigen] and edema toxin [composed of edema factor and protective antigen]) (*18*–*20*). The capsule prevents phagocytosis of the vegetative form of *B. anthracis* by macrophages, allowing it to evade the immune system. The two toxins also facilitate immune system evasion by disrupting various immune cell functions (e.g., cellular signaling and cell migration) (*21*,*22*). In addition, they impair macrophages, neutrophils, and dendritic cell functions (*23*,*24*) and inhibit host B- and T-cell immune responses (*25*). Lethal toxin causes apoptosis of endothelial cells within the vascular system and is thought to contribute to hemorrhage (*26*). These combined effects allow bacterial proliferation and lead to the high morbidity and mortality associated with anthrax. Historically, antiserum appeared to be an effective treatment. In aggregate data from the preantibiotic era, patients with cutaneous anthrax who were treated with antiserum had a substantially lower mortality rate than those who remained untreated (7.6% for patients who received antiserum during 1903−1941 compared with 23.7% for patients who did not receive antiserum during 1888−1920) (*27*).

Before the bioterrorism-related inhalation anthrax cases in 2001 (*13*), the anthrax mortality rate for the cases before the 1960s approached 90% for inhalation anthrax (*28*) and neared 100% for anthrax meningitis (*29*). Mortality rates have improved with advancements in critical care; however, even with treatment, mortality ranges from <2% for cutaneous anthrax (*30*), to 45% for inhalation anthrax (*28*), and to 92% for anthrax meningitis (*30*). In a mass-casualty event after a wide-area aerosol release of *B. anthracis* spores, mortality rates potentially could resemble those observed before the advent of modern critical care.

In 2013 and 2014, CDC published guidelines and recommendations for PEP and treatment of anthrax in pregnant and lactating persons (*31*), nonpregnant adults (*27*), and children (*32*). Those guidelines incorporated published and unpublished data from in vitro studies and U.S. inhalation anthrax cases since 2001 and also relied on expert opinion. In addition to addressing PEP and treatment of anthrax across all populations, those previously published guidelines described potentially beneficial critical care measures and clinical procedures for persons with anthrax, which are not addressed in this report. The ACIP recommendations for the use of anthrax vaccine remain unchanged (*33*). Vaccination of persons at risk for anthrax infection (e.g., travelers and laboratorians) can prevent disease. For unvaccinated or incompletely vaccinated persons exposed to *B. anthracis*, PEP includes both antimicrobial drugs with activity against *B. anthracis* (PEPAbx) and anthrax vaccine (PEPVx). This report updates best practices from previous guidelines for antimicrobial drug and antitoxin use for both PEP and treatment. The updated guidelines in this report are based on systematic reviews of the literature and associated data analyses regarding in vitro antimicrobial drug activity against *B. anthracis*; in vivo antimicrobial drug efficacy for PEPAbx and treatment; in vivo and human antitoxin efficacy for PEP, treatment, or both; and human survival after PEPAbx and treatment of localized anthrax, systemic anthrax, and anthrax meningitis. A review of safety information for the Food and Drug Administration (FDA)-approved antimicrobial drugs under consideration also served as a basis for the updated guidelines. Furthermore, these updated guidelines include new recommendations regarding special considerations for the diagnosis and treatment of anthrax meningitis, including comorbid, social, and clinical predictors of meningitis. This report recommends use of the following antimicrobial drugs that are not approved by FDA for anthrax PEPAbx or treatment: amoxicillin, amoxicillin/clavulanate, ampicillin/sulbactam, chloramphenicol, clarithromycin, clindamycin, dalbavancin, eravacycline, imipenem/cilastatin, linezolid, meropenem, moxifloxacin, ofloxacin, omadacycline, penicillin VK, piperacillin/tazobactam, rifampin, and vancomycin. Ciprofloxacin, doxycycline, levofloxacin, minocycline, penicillin G, and tetracycline are approved by FDA for anthrax PEPAbx, treatment, or both, but the specific uses (e.g., doses and dosing schedules) recommended in this report might differ from the FDA-approved labeling.

The intended audiences for the guidelines in this report are primary care providers and public health professionals. The antimicrobial drug, antitoxin, and other recommendations for PEP and treatment are best practices for the clinical management of single patient cases or limited outbreaks of naturally acquired anthrax. In addition, these guidelines address the issue of *B. anthracis* strains resistant to previously recommended antimicrobial drugs. Extensive antimicrobial drug options are provided for situations when first-line antimicrobial drugs are not tolerated or are contraindicated or after a bioterrorism event involving a wide-area aerosol release of *B. anthracis* spores over a heavily populated area. The latter might require a shift to crisis standards of care when supplies of first-line antimicrobial drugs have been depleted.

## Methods

To update the guidelines in this report, CDC established a CDC team to conduct systematic reviews and convened multiple work groups with external experts to review the data. CDC held meetings with the work groups to solicit information from individual members rather than obtain consensus from the whole group. After the review of the evidence and consideration of suggestions from members of the work group, CDC finalized the guidance and recommendations in this report. The composition and charge for each work group are described as follows:

- The CDC Anthrax Work Group Steering Committee, composed of CDC subject matter experts in anthrax, infectious diseases, pediatrics, obstetrics, and emergency response, identified other subject matter experts in anthrax, clinical medicine, microbiology, and pharmacology to participate in the Anthrax Work Group.
- The Anthrax Work Group was formed in December 2019 and included federal interagency and academic subject matter experts in anthrax, antimicrobial drugs, antitoxins, biopreparedness, microbiology, obstetrics and gynecology, pediatrics, infectious diseases, pharmacokinetics, pharmacodynamics, pharmacology, and public health. This work group reviewed the summarized systematic review data and commented on the recommendations derived from these data.
- The Anthrax Clinical Guidelines Team, composed of CDC staff members from the Division of High-Consequence Pathogens and Pathology, the Division of Preparedness and Emerging Infections, the Division of Birth Defects and Infant Disorders, and the Division of Human Development and Disability, convened and managed the Anthrax Work Group. In spring 2021, two additional work groups began to meet and address special antimicrobial drug selection and dosing consideration for their respective populations: the Pediatric Anthrax Work Group and the Obstetrics and Gynecology Anthrax Work Group. The Pediatric Anthrax Work Group included pediatricians, a neonatologist, pediatric infectious disease specialists, pharmacokinetics specialists, and biopreparedness experts. The Obstetrics and Gynecology Anthrax Work Group included obstetricians and gynecologists, pediatricians, and a neonatologist.

### Systematic Reviews

The data used to update the CDC anthrax clinical guidelines were based mainly on four systematic reviews of the literature performed by the Anthrax Clinical Guidelines Team. For each systematic review, database searches were supplemented with studies identified through hand searching of references and communication with additional subject matter experts. The data from all systematic and other reviews were abstracted and summarized for outcomes of interest.

The first systematic review was an all-language review of *B. anthracis* in vitro antimicrobial drug susceptibility studies published during 1947–2019 (*34*). For this systematic review, studies were considered for further assessment only if they reported *B. anthracis* minimum inhibitory concentration (MIC) values that were measured by using standard laboratory conventional broth microdilution, broth macrodilution, or agar dilution methods as described by the Clinical and Laboratory Standards Institute (CLSI) or European Committee on Antimicrobial Susceptibility Testing guidelines. Any study that reported MIC values for *B. anthracis* strains and cited agar-based gradient strip MIC testing (e.g., Etest and Lilofilchem) following the manufacturer’s instructions also was considered for further assessment. This review included antimicrobial drugs with IV, intramuscular (IM), or oral formulations that were both commercially available in the United States and approved by FDA (although not necessarily for anthrax PEPAbx or treatment). The second all-language systematic review summarized antimicrobial drugs used in animal studies for PEPAbx or treatment of experimental infections with virulent *B. anthracis* published during 1947–2019 (*35*). The third systematic review updated a previous in vivo and clinical systematic review of anthrax antitoxin for treatment of inhalation anthrax (*36*) and was restricted to English-language articles published during 2015–2019 (*37*). In contrast to the original systematic review (*36*) that focused on antitoxin use for inhalation anthrax, this systematic review also included database searches from date of inception through 2014 for articles on PEP for animals and treatment of humans with systemic noninhalation anthrax.

The fourth systematic review was restricted to articles describing patients hospitalized for anthrax published in English during 1880–2018 (*38*). All human data were observational in nature. Cases were categorized into localized versus systemic disease. “Systemic illness” included evidence of organ damage or any of the following signs: hyperthermia or hypothermia, tachycardia, tachypnea, hypotension, or leukocytosis or leukopenia (*30*). Sensitivity analyses were performed when necessary to assess whether heterogeneity of case report quality affected results. Subsets of the data from this systematic review were analyzed for clinical (*38*), treatment (*39*), and cutaneous anthrax outcomes (*40*) and for the development of an anthrax meningitis screening tool (*41*).

A full systematic review of safety information was not conducted for two reasons. First, the antimicrobial drugs being considered were FDA approved, commercially available, and had safety profiles that were based on clinical usage data. Second, safety reviews of certain antimicrobial drugs had been conducted for the 2014 anthrax and 2021 plague guidelines. Instead, existing drug safety data sources were reviewed for 24 selected antimicrobial drugs under consideration for PEPAbx or treatment of anthrax, or both (*42*). Data from these tertiary sources were supplemented through focused PubMed searches. For antimicrobial drugs FDA approved before 2010, literature published in English during January 2018–April 2021 was identified via PubMed and reviewed for randomized controlled trials (RCTs) describing serious or severe adverse events (AEs) in adults, pregnant and lactating persons, and children. The criteria used to identify the severe or serious AEs for inclusion in the safety review of selected antimicrobials are defined in a previous publication (i.e., severe or serious AEs reported in the literature as possibly related, probably related, related, or definitely related to the study treatment, or if causality was not reported and causality could not be obtained from the authors) (*42*). For antimicrobial drugs that were FDA approved in 2010 or later, English-language literature identified in PubMed from inception through April 2021 was reviewed for all study types (i.e., RCTs, observational studies, and case series or case reports) describing serious or severe AEs in adults, pregnant and lactating persons, and children. Because of the limited data available in tertiary data sources for antimicrobial drugs FDA approved in 2010 or later, no date restrictions were applied to these literature searches. In addition, PubMed search terms captured all literature related to antimicrobial drug use in pregnant and lactating persons and children and were not specifically limited to serious or severe AEs.

Univariate and multivariable logistic regression was used to calculate odds ratios (ORs) with 95% CIs comparing the odds of survival between different treatments. For the in vivo systematic review (*35*), data were extracted from independent studies and combined into meta-analyses when multiple studies evaluated the same antimicrobial drugs. These data were used to estimate an overall OR and 95% CI for successful therapy. Data analyses were performed in SAS (version 9.4; SAS Institute), and p<0.05 was considered statistically significant. Pharmacokinetic/pharmacodynamic (PK/PD) analyses (i.e., Monte Carlo simulations by predicting the drug concentration-time profiles of 1,000 virtual patients for each drug and regimen) (*43*,*44*) were performed for in vivo animal efficacy studies of antimicrobial drugs when sufficient data were available.

### Review of the Evidence

The Anthrax Clinical Guidelines Team summarized systematic review data for review by the Anthrax Work Group during twice-monthly meetings held during December 2019–June 2021. In June 2021, CDC held 3 days of virtual meetings that included members of the Anthrax Work Group plus additional experts in anthrax, infectious diseases, pediatrics, obstetrics, pharmacology, and emergency response to review available scientific information and provide individual-level input on proposed CDC updates to previously published guidelines for prevention and treatment of anthrax. Additional specialties represented included biostatistics, critical care, emergency medicine, geriatrics, neurocritical care, neurosurgery, and preventive medicine. All internal CDC staff members involved in developing the guidelines and external consultants providing individual input submitted a written financial disclosure statement reporting any potential conflicts of interest related to questions discussed during the consultations or concerns involved in developing the updated CDC anthrax guidelines. The Anthrax Work Group co-chairs reviewed each reported association for potential competing interests and determined the appropriate action, as follows: disqualification from the panel, disqualification/recusal from topic review and discussion, or no disqualification needed. A competing interest was defined as any direct financial interest related to a product addressed in the section of the guidelines to which a panel member contributed content. Financial interests included direct receipt by the panel member of payments, gratuities, consultancies, honoraria, employment, grants, support for travel or accommodation, or gifts from an entity having a commercial interest in that product. Financial interest also included direct compensation for membership on an advisory board, data safety monitoring board, or speaker bureau. Compensation and support filtered through a panel member’s university or institution (e.g., grants or research funding) was not considered a competing interest. All federal employees, including CDC staff members, are subject to the Standards of Ethical Conduct for Employees of the Executive Branch (*45*).

The meeting attendees listened to presentations on anthrax clinical features (*38*), optimal antimicrobial drug treatment and PEP from both an efficacy (in vitro, in vivo, and observational clinical data) and safety standpoint (*34*,*35*,*39*,*42*,*46*,*47*), the value and use of antitoxins (*37*,*39*,*48*–*50*), and methods to rapidly identify and optimally treat anthrax meningitis (*39*–*41*,*51*,*52*). The attendees then provided individual expert opinions on proposed edits to previous antimicrobial drug and antitoxin PEP and treatment guidelines.

When evaluating the benefit versus harm of antimicrobial drugs for anthrax (PEP, treatment, or both), risks common to all antimicrobial drugs (e.g., hypersensitivity, *Clostridioides difficile* infection and associated diarrhea, and selective pressure for colonization and subsequent infection by resistant organisms) were considered in the risk-benefit evaluation. Additional considerations included specific AEs for each antimicrobial drug and antimicrobial drug class and patient- and population-specific characteristics that influence antimicrobial drug selection (e.g., renal function, drug allergies, and interacting concomitant medications).

Individual expert opinions on prophylaxis and treatment options were collated for later internal CDC discussions. After the meeting, the Anthrax Clinical Guidelines Team drafted narrative summaries, recommendations, and tables. The guideline authors shared the draft guidelines with the anthrax work groups and refined the proposed changes to the guidelines based, in part, on their feedback. This report summarizes the presentations and discussions from the meeting and updates previous CDC guidelines for prevention and treatment of anthrax.

## Summary of Key Findings

### In Vitro *B. anthracis* Antimicrobial Drug Susceptibility Testing

A systematic review identified 105 sources describing in vitro *B. anthracis* antimicrobial drug susceptibility testing representing results for 169 different compounds (*34*). After articles were excluded for incomplete MIC data or nonstandard methodology, 39 remained that contained data on 43 antimicrobial drugs of interest. Except for aztreonam, ceftriaxone, and trimethoprim/sulfamethoxazole, most antimicrobial drugs appeared useful per the CLSI guidelines (i.e., had low MICs for *B. anthracis* in comparison with clinically relevant unbound drug concentrations) (Table 1). Omadacycline was identified as a potentially effective option for certain tetracycline-resistance mechanisms (e.g., efflux pumps) because unpublished in vitro data have indicated it might evade these mechanisms. Naturally occurring high-level resistance was observed in multiple studies of penicillin-class antimicrobial drugs, eight of eight studies of a representative cephalosporin (i.e., ceftriaxone), and five of five studies of trimethoprim/sulfamethoxazole.

**TABLE 1:** Minimum inhibitory concentration data from the antimicrobial study that evaluated the most *Bacillus anthracis* strains, by antimicrobial drug

Antimicrobial drug	No. of studies analyzed	MIC_50_ (*µ*g/mL)	MIC_90_ (*µ*g/mL)	No. of wildtype strains
Ciprofloxacin*	28	0.03	0.03	110
Delafloxacin (ABT-492)	1	0.063	0.063	27
Levofloxacin*	8	0.12	0.25	96
Moxifloxacin*	6	0.12	0.5	51
Ofloxacin	3	0.25	0.25	96
Doxycycline*	18	0.015	0.03	110
Minocycline	3	—^†^	—^†^	3
Omadacycline	1	0.03	0.06	30
Tetracycline*	13	0.06	0.12	110
Tigecycline	2	≤0.12	0.25	53
Amoxicillin*	10	0.06	0.06	110
Amoxicillin/clavulanate*	7	0.032/0.016	0.047/0.024	48
Ampicillin	4	0.03	0.03	22
Ampicillin/sulbactam*	1	0.015/—^§^	0.015/—^§^	22
Oxacillin	2	≤0.25	≤0.25	18
Penicillin*	21	0.015	0.03	110
Piperacillin	3	1	1	96
Piperacillin/tazobactam	1	≤2/4	≤2/4	28
Imipenem	3	0.12	0.12	96
Meropenem	7	0.12	4^¶^	28
Amikacin	3^**^	0.5	1	30
Gentamicin	15	0.12	0.25	110
Neomycin	1	—^†^	—^†^	7
Streptomycin	7	0.5	1	110
Tobramycin	2	0.25	1	22
Daptomycin	3	2	4	30
Dalbavancin	1	0.06	0.25	30
Oritavancin	1	0.5	2	30
Vancomycin*	14	2	2	110
Fosfomycin	2	—^†^	—^†^	3
Rifampin	6	0.12	0.25	110
Clindamycin*	12	0.12	0.25	110
Lincomycin	1	—^†^	—^†^	3
Azithromycin	5	3	6	73
Clarithromycin*	5	0.12	0.12	27
Erythromycin	14	0.12	0.25	110
Linezolid*	5	1	2	110
Chloramphenicol	9	4	4	110
Quinupristin/dalfopristin	2	0.5	1	18
Metronidazole	1	—^†^	—^†^	3
Aztreonam^††^	2	128	128	96
Ceftriaxone^††^	8	16	16	110
Trimethoprim/sulfamethoxazole^††^	5	>4/76	>4/76	96

**Source:** Maxson T, Kongphet-Tran T, Mongkolrattanothai T, et al. Systematic review of in vitro antimicrobial susceptibility testing for *Bacillus anthracis*, 1947–2019. Clin Infect Dis 2022;75(Suppl 3):S373–8.

**Abbreviations:** MIC_50_ = minimum inhibitory concentration required to inhibit the growth of 50% of isolates tested; MIC_90_ = minimum inhibitory concentration required to inhibit the growth of 90% of isolates tested.

* MIC_50_ and MIC_90_ values from studies of ciprofloxacin, levofloxacin, moxifloxacin, clarithromycin, penicillin, amoxicillin, amoxicillin/clavulanate, ampicillin/sulbactam, doxycycline, tetracycline, linezolid, clindamycin, and vancomycin align with findings of an unpublished CDC study of 50 wildtype *Bacillus anthracis* strains by conventional broth microdilution. The CDC MIC_50_ and MIC_90_ values were within ±1 doubling dilution concentration to those listed.

^†^ MIC_50_ and MIC_90_ values were omitted if <10 strains were tested.

^§^ Sulbactam concentration was not detailed in the source study.

^¶^ In an unpublished CDC study of 50 *B. anthracis* strains, the MIC_90_ value for meropenem was closer to the MIC_50_ value (0.6 *µ*g/mL meropenem) reported in a previous study (**Source:** Cavallo JD, Ramisse F, Girardet M, Vaissaire J, Mock M, Hernandez E. Antibiotic susceptibilities of 96 isolates of *Bacillus anthracis* isolated in France between 1994 and 2000. Antimicrob Agents Chemother 2002;46:2307–9).

** Includes unpublished study findings.

^††^ Data demonstrate intrinsic resistance in *B. anthracis*.

### In Vivo PEPAbx and Treatment of Anthrax

A systematic review identified 62 sources on in vivo PEPAbx and treatment of anthrax that described approximately 800 study arms with approximately 12,000 animals (*35*). After exclusions, data were analyzed from 37 sources that described 33 antimicrobial drugs and 309 study arms with 3,450 animals, including 1,423 mice, 807 rabbits, 660 hamsters, 329 nonhuman primates, and 231 guinea pigs. Antimicrobial drugs expected to be effective for PEPAbx or treatment of anthrax were identified by examining the odds of survival for selected antimicrobial drugs compared with untreated or ciprofloxacin- or doxycycline-treated controls (*35*) (Table 2). Efficacy of clinically relevant dosage regimens was predicted for antibiotics with sufficient PK and anthrax PEPAbx or treatment efficacy data.

**TABLE 2:** Odds of survival from in vivo studies of prophylaxis against *Bacillus anthracis* infection or treatment for anthrax, by antimicrobial drug

Antimicrobial drug	Drug class	Mechanism of action	PEPAbx or Rx	No. of studies	OR (95% CI) vs. no treatment	No. of studies	OR (95% CI) vs. ciprofloxacin	No. of studies	OR (95% CI) vs. doxycycline
Ciprofloxacin	Fluoroquinolone	C	PEPAbx	10	11.5 (5.2–25.3)*	NA	NA	3	1.1 (0.7–1.8)
Levofloxacin	Fluoroquinolone	C	PEPAbx	4	124.8 (0.4–42,865)	1	2.2 (0.2–29.8)	NA	NA
Ofloxacin^†^	Quinolone	C	PEPAbx	1	171 (7.5–3,910)*	NA	NA	NA	NA
Amoxicillin	Penicillin	C	PEPAbx	1 1	357 (6.4–19,943)* 1,345 (76.2–23,762)*^,§^	NA	NA	NA	NA
Penicillin	Penicillin	C	PEPAbx	2	4.1 (0.9–19.9)	NA	NA	NA	NA
Procaine penicillin G^¶^	Penicillin	C	PEPAbx	2	8.2 (0–5,846,972)	1	0.3 (0–3.5)	1	0.3 (0–3.1)
Amoxicillin/clavulanate	Penicillin/ß-lactamase inhibitor	C	PEPAbx	1 1 1	187 (3.2–10,884)* 45.0 (0.7–3,042) 1,617 (91.7–24,498)*^,§^	NA	NA	NA	NA
Dalbavancin**	Glycopeptide	C	PEPAbx	1	3,381 (63.7–179,559)*	NA	NC^††^	NA	NA
Oritavancin**	Glycopeptide	C	PEPAbx	1	46.3 (2.6–816)*	1	0.1 (0–0.8)*	NA	NA
Daptomycin**	Lipopeptide	C	PEPAbx	1	81.0 (4.4–1,505)*	1	1.9 (0.2–21.5)	NA	NA
Doxycycline	Tetracycline	S	PEPAbx	5	5.4 (3.2–9.0)*	3	0.9 (0.6–1.3)	NA	NA
Minocycline	Tetracycline	S	PEPAbx	1	1,489 (84.4–26,267)*	NA	NA	1	99.0 (5.9–1,651)*
Omadacycline	Tetracycline	S	PEPAbx	1	50.4 (13.9–182)*	1	0.2 (0–3.6)	1	1.1 (0.4–3.1)
Tetracycline	Tetracycline	S	PEPAbx	1	17.9 (7.8–40.9)*	NA	NA	1	1.1 (0.6–2.0)
Azithromycin	Macrolide	S	PEPAbx	1 1	25.0 (1.1–562.8)* 7.5 (0.3–186)^§^	NA	NA	NA	NA
Clarithromycin	Macrolide	S	PEPAbx	1	16.2 (0.6–441.7)^††^	NA	NA	NA	NA
Erythromycin ethylsuccinate^§§^	Macrolide	S	PEPAbx	1	21.0 (1.0–454)	NA	NA	NA	NA
Imipenem^¶¶,^***	Carbapenem	C	PEPAbx	1	29.9 (1.3–692)*	NA	NA	NA	NA
Cefazolin***^,†††^	Cephalosporin	C	PEPAbx	1	8.4 (0.4–177)	NA	NA	NA	NA
Gentamicin***^,†††^	Aminoglycoside	C	PEPAbx	1	3.0 (0.1–83.4)	NA	NA	NA	NA
Chloramphenicol**^,§§^	Amphenicol	S	PEPAbx	1	10.2 (0.6–184)	NA	NA	NA	NA
Trimethoprim/sulfamethoxazole***	Sulfonamide	S	PEPAbx	1	15.4 (0.8–305)	NA	NA	NA	NA
Ciprofloxacin	Fluoroquinolone	C	Rx	7	12.5 (3.9–40.5)*	NA	NA	2	0.8 (0.6–1.2)
Levofloxacin	Fluoroquinolone	C	Rx	4	337 (0.5–210,268)^§§§^	NA	NA	NA	NA
Imipenem^¶¶^	Carbapenem	C	Rx	1	63.0 (2.5–1,558)*	NA	NA	NA	NA
Penicillin	Penicillin	C	Rx	1	24.1 (1.0–567)^††^	NA	NA	NA	NA
Procaine penicillin^¶^	Penicillin	C	Rx	1	24.1 (1.0–567)*	NA	NA	NA	NA
Amoxicillin/clavulanate^¶¶¶^	Penicillin/ß-lactamase inhibitor	C	Rx	1	25.0 (1.1–563)*	NA	NA	NA	NA
Vancomycin	Glycopeptide	C	Rx	1	52.7 (2.3–1,211)*	NA	NA	NA	NA
Dalbavancin	Glycopeptide	C	Rx	1	138 (6.8–2,780)*	1	0.7 (0–15.9)	NA	NA
Oritavancin	Glycopeptide	C	Rx	1	39.0 (2.1–734)*	1	0.3 (0.1–1.1)	NA	NA
Doxycycline	Tetracycline	S	Rx	4	4.9 (2.1–11.6)*	1	1.6 (0.9–2.8)	NA	NA
Eravacycline	Tetracycline	S	Rx	1	369 (6.4–21,191)*	NA	NA	NA	NA
Omadacycline	Tetracycline	S	Rx	1	9.8 (1.9–50.6)*	1	0.4 (0.1–3.2)	1	0.6 (0.1–4.1)
Clindamycin	Lincosamide	S	Rx	1	24.4 (1.0–581)*	NA	NA	NA	NA
Clarithromycin^¶¶¶^	Macrolide	S	Rx	1	5.5 (0.3–111)	NA	NA	NA	NA
Linezolid	Oxazolidinone	S	Rx	1	17.2 (0.8–381)	NA	NA	NA	NA
Rifampin	Rifamycin	C	Rx	1	3.4 (0.2–74.4)	NA	NA	NA	NA

**Source:** Kennedy JL, Bulitta JB, Chatham-Stephens K, et al. Postexposure prophylaxis and treatment of *Bacillus anthracis* infections: a systematic review and meta-analyses of animal models, 1947–2019. Clin Infect Dis 2022;75(Suppl 3):S379–91.

**Abbreviations:** C = bactericidal; IM = intramuscular; IV = intravenous; NA = not applicable; NC = not calculated; OR = odds ratio; PEPAbx = antimicrobial postexposure prophylaxis for anthrax; Rx = treatment; S = bacteriostatic.

* Statistically significant (p = 0.05).

^†^ The study using this antimicrobial drug used an intranasal challenge route and a vaccine.

^§^ Individual ORs are shown when studies had zero cells and could not be combined.

^¶^ Only available in the United States as an IM formulation for humans, although animals might have received other formulations or routes of administration.

** Only available in the United States as an IV formulation for humans, although animals might have received other formulations or routes of administration.

^††^ At least one study was removed to allow OR calculation.

^§§^ The study using this antimicrobial drug used an intraperitoneal challenge route.

^¶¶^ Only available in the United States as an IV formulation in combination with cilastatin for humans. Animals received only imipenem, which might have used another route of administration.

*** The study using this antimicrobial drug used an intranasal challenge route.

^†††^ Only available in the United States as an IM or IV formulation for humans, although animals might have received other formulations.

^§§§^ High heterogeneity score.

^¶¶¶^ Only available in the United States as an oral formulation for humans, although animals might have received other formulations or routes of administration.

#### PEP

 Meta-analyses and most individual animal studies that could not be combined into any of the meta-analyses found a survival benefit for PEPAbx compared with no PEPAbx for amoxicillin, amoxicillin/clavulanate (two of three studies), ciprofloxacin, dalbavancin, daptomycin, doxycycline, imipenem, minocycline, ofloxacin, omadacycline, oritavancin, and tetracycline. Of four studies on macrolide-class antimicrobial drugs, only one study that evaluated azithromycin demonstrated a benefit. No statistically significant benefit was identified for cefazolin, chloramphenicol, gentamicin, levofloxacin, penicillin, and trimethoprim/sulfamethoxazole when used in a PEPAbx model. When compared with positive controls, minocycline performed better than doxycycline, and oritavancin performed less well than ciprofloxacin. Included PEPAbx studies used aerosol exposures, except for those using cefazolin, gentamicin, imipenem, ofloxacin, and trimethoprim/sulfamethoxazole (which used intranasal challenges) and those that included chloramphenicol and erythromycin ethylsuccinate (which used intraperitoneal challenges). For PEPAbx against naturally occurring *B. anthracis* infection in humans, Monte Carlo simulations predicted that unbound drug exposures adequately covered the MICs required to inhibit the growth of 90% of organisms (MIC_90_) for ciprofloxacin, doxycycline, and levofloxacin; dalbavancin only covered the MICs required to inhibit the growth of 50% of organisms (MIC_50_)_._

#### Treatment

 The in vivo meta-analyses and studies that could not be combined into any of the meta-analyses demonstrated a survival benefit for monotherapy compared with no treatment for amoxicillin/clavulanate, ciprofloxacin, clindamycin, dalbavancin, doxycycline, eravacycline, imipenem, omadacycline, oritavancin, penicillin, and vancomycin. Clarithromycin, levofloxacin, linezolid, and rifampin did not demonstrate a statistically significant survival benefit when compared with no treatment. One rabbit model for anthrax meningitis indicated a survival benefit for amoxicillin/clavulanate and ampicillin with or without sulbactam. A second rabbit model for anthrax meningitis demonstrated a survival benefit for clindamycin. For treatment of naturally occurring anthrax in humans, Monte Carlo simulations predicted that unbound drug exposures adequately covered the MIC_90_ for ciprofloxacin, doxycycline, and levofloxacin (JS Bradley, MD, University of California San Diego School of Medicine, JB Bulitta, PhD, University of Florida College of Pharmacy, personal communication, November 2022).

### Antitoxins for PEP and Treatment of Anthrax

A systematic review identified 757 sources that reported on use of anthrax antitoxins in prevention or treatment of systemic anthrax disease (*37*). After screening the titles and abstracts for relevant articles and applying exclusion criteria, data were abstracted from 14 papers.

#### PEP

 Animal data indicated that PEP with any of the anthrax antitoxins (i.e., anthrax immunoglobulin intravenous [AIGIV; polyclonal] and obiltoxaximab and raxibacumab [both monoclonal]) provided a statistically significant survival benefit (p<0.04 for all three antitoxins) compared with no treatment, with the earliest administration after exposure demonstrating the greatest benefit (*53*–*56*).

#### Coadministration With Anthrax Vaccine Adsorbed

 Although data indicated that the polyclonal antitoxin AIGIV should not be coadministered with anthrax vaccine adsorbed (AVA) (*57*), noninterference was demonstrated between raxibacumab and AVA, allowing their coadministration (*58*). No data were available on coadministration of obiltoxaximab and AVA.

#### Adjunctive Treatment

 A head-to-head comparison of the three antitoxins for treatment after aerosol exposure to *B. anthracis* in an animal model demonstrated a survival benefit compared with placebo. No differences were observed in survival between the monoclonal antitoxins obiltoxaximab and raxibacumab, but both monoclonals were considerably more effective than the polyclonal antitoxin AIGIV (*50*). However, this animal model did not use the highest FDA-approved AIGIV dose. The higher dose might have lessened the survival difference for the monoclonal antitoxins compared with the polyclonal antitoxin (*37*).

In animal models, treatment with the polyclonal or either monoclonal antitoxin provided a statistically significant (p≤0.0001for all three antitoxins) survival benefit compared with no treatment. However, adding the polyclonal antitoxin or either monoclonal antitoxin to antimicrobial drugs did not significantly improve survival over antimicrobial drugs alone (*59*–*61*).

### Human Data for PEPAbx of Anthrax

In an observational study conducted in the former Soviet Union, persons exposed to anthrax-affected animals were administered PEPAbx with a penicillin or tetracycline (*62*). After noninhalation exposure, 17% of 339 persons who did not receive PEPAbx developed anthrax. In contrast, only 1.7% of 287 persons who received a short course (i.e., 3 days) of PEPAbx developed anthrax (p<0.001). After this finding, when various PEPAbx regimens were used in 407 persons exposed to *B. anthracis,* none developed anthrax.

### Human Clinical and Treatment Data for Anthrax

A systematic review identified 13,082 sources of human clinical and treatment data on patients hospitalized for anthrax (*30*,*38*). After removal of duplicates and review of titles and abstracts for relevance, 952 full-text articles were reviewed; 584 were excluded because they lacked data on patients with confirmed anthrax who were hospitalized or died. The remaining 368 articles (composed of case reports, case series, and line lists) yielded 965 adult and pediatric patients with confirmed anthrax who were hospitalized or died. Two separate analyses were performed on this data set of 965 patients, one focused on the clinical aspects of anthrax (*38*) and the other on treatment (*39*).

Therapies evaluated in the treatment systematic review (*39*) included antimicrobial drugs, antiserum/antitoxin, steroids, and mannitol. To analyze antimicrobial drug use during the era of modern clinical care, the database was restricted to adult cases published during 1940–2018 (N = 303). Analyses of adult cases were restricted to 1900–2018 (N = 422) for antiserum/antitoxin use, 1950–2018 (N = 253) for steroid use, and 1960–2018 (N = 232) for mannitol use.

#### Treatment in Hospitalized Adults

 In adult patients hospitalized for anthrax, monotherapy, including penicillin monotherapy, resulted in a high survival rate (98%) for those with localized cutaneous anthrax (*39*). For adults with systemic cutaneous anthrax without meningitis, survivorship was high if they received any treatment; only one patient in this category died. Survival with penicillin monotherapy was 89% for those with systemic illness from any route if they did not have meningitis. Adults with inhalation anthrax without meningitis fared poorly with monotherapy; only 17% (one of six) survived compared with 70% (seven of 10) with combination therapy. For anthrax meningitis, neither antimicrobial drug monotherapy nor combination therapy was particularly effective; only 21% (three of 14) survived with monotherapy and 17% (three of 18) with combination therapy. However, both monotherapy and combination therapy were more effective than no therapy.

Multivariable analysis of survival for systemically ill patients administered specific therapies suggested effective treatment options (*39*) (Table 3). After controlling for shock and altered mental status, odds of survival among systemically ill patients with anthrax were higher for those receiving bactericidal antimicrobial drugs alone compared with protein synthesis inhibitors (PSIs) alone (p = 0.047). In addition, when controlling for hypoxia, shock, and altered mental status (i.e., illness severity), odds of survival were not improved by adding a PSI to a bactericidal antimicrobial drug. Odds of survival also were not improved by adding a second class of antimicrobial drug or an antitoxin to an antimicrobial drug.

**TABLE 3:** Survival for adults hospitalized with systemic anthrax, by specified treatment* comparisons, adjusted for illness severity — 1940–2018

Treatment comparison	No. of patients	Survived No. (%)	No. of patients	Died No. (%)	Multivariable analysis		Variables analyzed		
					OR (95% CI)	p value^†^	Hypoxia^§^	Shock^¶^	AMS
Bactericidal drug(s) alone vs. PSI(s) alone	66	60 (91)	33	27 (82)	4.57 (1.02–20.47)	0.047	No	Yes	Yes
Bactericidal drug(s) and PSI(s) vs. bactericidal drug(s) alone	86	26 (30)	41	14 (34)	1.54 (0.52–4.50)	0.43	Yes	Yes	Yes
Bactericidal drug(s) and PSI(s) vs. PSI(s) alone	32	26 (81)	20	14 (70)	3.60 (0.73–17.64)	0.11	Yes	No	No
Bactericidal drug(s) and PSI(s) vs. bactericidal drug(s) alone or PSI(s) alone	92	26 (28)	47	14 (30)	1.89 (0.67–5.31)	0.23	Yes	Yes	Yes
Antimicrobial drug combination therapy vs. monotherapy**	87	31 (36)	47	25 (53)	0.88 (0.33–2.35)	0.80	Yes	Yes	Yes
Antimicrobial drug(s) and antitoxin/antiserum vs. antimicrobial drug(s)	100	7 (7)	55	5 (9)	1.31 (0.24–7.07)	0.75	Yes	Yes	Yes

**Source:** Person MK, Cook R, Bradley JS, Hupert N, Bower WA, Hendricks K. Systematic review of hospital treatment outcomes for naturally acquired and bioterrorism-related anthrax, 1880–2018. Clin Infect Dis 2022;75(Suppl 3):S392–401.

**Abbreviations:** AMS = altered mental status; OR = odds ratio; PSI = protein synthesis inhibitor.

* Patients are only included in each row if they had the specified treatment being compared. Patients are considered to have the specified treatment if administered in the first 2 days of hospitalization. Patients are classified as having no antimicrobial drug treatment if they did not receive any antimicrobial drugs throughout hospitalization. Patients were excluded from analysis if they were dead on arrival, died on day 1 of hospitalization, or if survival status was unknown. Patients who were administered antimicrobial drugs appropriate for anthrax treatment before hospitalization also were excluded. Sulfa drugs and cephalosporins are not considered appropriate for anthrax treatment and therefore did not contribute to the count of antimicrobial drugs. Patients who were administered antitoxin/antiserum at any time in the course of their treatment were excluded except when comparing antimicrobial drugs and antitoxin/antiserum to antimicrobial drugs alone. Certain patients were excluded because of missing sign, symptom, and treatment data (**Source:** Meselson M, Guillemin J, Hugh-Jones M, et al. The Sverdlovsk anthrax outbreak of 1979. Science 1994;266:1202–8).

^†^ p value calculated by Wald chi-square; p<0.05 was considered statistically significant.

^§^ Ventilated or intubated or respiratory rate >30 breaths per minute.

^¶^ Administered vasopressors or systolic blood pressure <90 mm Hg.

** Included one anthrax meningitis patient whose illness did not meet the definition of systemic.

Survivorship was compared in steroid recipients and nonrecipients with various complications. For patients with shock, survivors included zero of two (0%) recipients and six of 17 (35%) nonrecipients. For patients with head or neck involvement, survivors included four of seven (57%) recipients and 38 of 45 (84%) nonrecipients. For those with edema involving more than one extremity, survivors included four of eight (50%) recipients and 24 of 29 (83%) nonrecipients. Survivorship generally was higher among nonrecipients, suggesting steroids were not beneficial in these specific risk groups.

Antitoxins with or without antimicrobial drugs at any point throughout hospitalization could not be demonstrated to improve outcomes. After controlling for age and illness severity, the odds of survival were not different for recipients compared with nonrecipients (adjusted OR = 1.49; 95% CI = 0.73–3.05).

#### Complications

 Multiple clinically relevant complications might occur in association with anthrax (*38*). Sepsis developed in approximately 70% of patients with systemic disease. Approximately one fourth of adults hospitalized with systemic cutaneous or ingestion anthrax and one third with inhalation anthrax developed secondary meningitis. Evidence of coagulopathy was reported in approximately one third of adults with ingestion, inhalation, and injection anthrax and those with primary meningitis. Arrhythmias occasionally were observed, most commonly in adults with inhalation anthrax (7%). Respiratory failure requiring ventilation often occurred in adults with injection anthrax (32%) and inhalation anthrax (23%). Severe disease was associated with diabetes, obesity, hypertension, and chronic obstructive pulmonary disease (p<0.01 for all) in patients with cutaneous anthrax (*50*).

Similar to adults, complications of anthrax in children varied by site of initial infection (*38*). Sepsis developed in 60%–84% of children with inhalation, ingestion, and systemic cutaneous anthrax. Secondary meningitis was observed in 25% of children with systemic disease associated with cutaneous anthrax, 40% with inhalation anthrax, and 30% with ingestion anthrax. Coagulopathy occurred in approximately 40% of inhalation and ingestion cases.

Many adults developed clinically relevant fluid collections (*38*). Although pleural effusion was most commonly a complication of inhalation anthrax (76%), it also occurred in 3%–10% of patients with ingestion, systemic cutaneous, and injection anthrax and primary anthrax meningitis. In 2006 and 2011, spectrographic analysis of pleural fluid from patients with inhalation anthrax demonstrated the presence of anthrax lethal factor, one of the three protein components of anthrax toxin. In both patients, lethal factor levels in the pleural fluid exceeded concurrently measured levels in plasma and serum (*63*,*64*). Ascites was observed in 52% of adults with ingestion anthrax; in 4% of those with inhalation anthrax; and in one person each with injection anthrax, systemic cutaneous anthrax, and primary anthrax meningitis (*38*). Ascites also is thought to serve as a reservoir for lethal factor. Pericardial effusions were noted in 21% of patients with inhalation anthrax.

#### Survival and Length of Stay

During 1940–2018, the mortality rates in adults were 92% (22 of 24) for primary anthrax meningitis, 75% (36 of 48) for inhalation anthrax, 72% (23 of 32) for ingestion anthrax, 33% (19 of 58) for injection anthrax, and 22% (45 of 206) for cutaneous anthrax. In children, the mortality rates were 100% (two of two) for primary anthrax meningitis, 100% (two of two) for inhalation anthrax, 48% (11 of 23) for ingestion anthrax, and 8% (nine of 110) for cutaneous anthrax.

During 1940–2018, adults who died tended to do so quickly, with a median hospital stay of only 1 day (IQR = 1–3 days), whereas those who survived had a median stay of 14 days (IQR = 9–22 days). Among adults who survived, median stays for those with localized cutaneous anthrax were 11 days (IQR = 8–16 days); systemic anthrax without meningitis, 16 days (IQR = 10–29 days); and with meningitis, 19 days (IQR = 12–32 days) (*39*).

### Studies of Hospitalized Patients with Cutaneous Anthrax

#### Antimicrobial Drug Selection

 In a case series from the Kyrgyz Republic, 230 patients were hospitalized for cutaneous anthrax during 2005–2012; all 167 with mild illness and 43 of 44 with moderate illness received monotherapy and survived (*52*). The remaining patient with moderate illness and all 19 patients with severe illness received dual therapy and survived. No patient received triple therapy.

#### Signs and Symptoms Predictive of Fatal Outcome

 A systematic review of reported cases among adults hospitalized for cutaneous anthrax during 1950–2018 found that presenting clinical features significantly associated with overall mortality on univariate analysis included constitutional symptoms (e.g., fever, chills, and anxiety), specific dermatologic issues (e.g., skin trauma, thoracic edema, and malignant pustule edema), diastolic hypotension, nausea and vomiting, headache and other neurologic signs (e.g., cranial nerve and other focal signs and seizures), and evidence of a coagulopathy (p<0.05) (*40*). Lymphadenopathy was associated with fatal outcomes within the first 3 days of hospitalization, and abdominal pain was associated with later fatal outcomes. Bacteremia noted at any point throughout hospitalization was associated with overall mortality.

### Special Considerations for the Diagnosis and Treatment of Anthrax Meningitis

#### Comorbidities and Social History Predictive of Meningitis

 In the Kyrgyz Republic case series, 126 of the 230 patients with cutaneous anthrax could be categorized by a clinical (i.e., signs and symptoms) algorithm into likely or not likely meningitis (*52*). Obesity, diabetes, hypertension, and chronic obstructive pulmonary disease were associated with likely meningitis. In addition, former and current smoking and former alcohol use were associated with likely meningitis.

#### Signs and Symptoms Predictive of Meningitis

 Three recent studies (two of which shared overlapping data sets) have examined presenting signs, symptoms, and laboratory results associated with anthrax meningitis (*40*,*41*,*52*) (Table 4). One study evaluated systemic anthrax in adults (*41*) and two evaluated cutaneous anthrax in adults (*40*,*52*). All three studies found that fever and chills, nausea and vomiting, and headache were associated with meningitis. Two studies also found nonheadache, nonmeningeal neurologic signs (e.g., seizures and cranial nerve signs) to be associated with meningitis. Predictors found in one study included altered mental status and meningeal signs, fever (>38.0°C or >100.4°F), tachypnea (>20 breaths per minutes), tachycardia (≥100 beats per minute), systolic and diastolic hypertension (≥130 and ≥90 mm Hg, respectively), and evidence of coagulopathy. Presenting bacteremia was predictive of meningitis in one of the studies and bacteremia at any point throughout hospitalization was predictive in another.

**TABLE 4:** Presenting signs, symptoms, and laboratory test results associated with meningitis for adults with systemic or cutaneous anthrax

Symptom/Sign/Laboratory test result	Patient population		
	Systemic anthrax*^,†^ (N = 419)	Cutaneous anthrax^†,§^ (N = 182)	Cutaneous anthrax^¶^ (N = 126)
**Time frame**	1880–2018	1950–2018	2005–2012
**Symptom**			
Fever/chills	X	X	X
Nausea/vomiting	X	X	X
Headache	X	X	X
Abdominal pain	ANA	X	ANA
**Sign**			
Evidence of coagulopathy (i.e., bleeding)	ANA	X	NE
**Vital sign**			
Fever (>38.0^°^C)	ANA	ANA	X
Tachycardia (>90 beats per minute)	NE	ANA	NE
Tachycardia (≥100 beats per minute)	ANA	NE	X
Tachypnea (>20 breaths per minute)	ANA	ANA	X
Systolic hypertension (≥130 mm Hg)	NE	NE	X
Diastolic hypertension (≥90 mm Hg)	NE	NE	X
Fulminant	X	NE	NE
**Dermatologic**			
Lymphadenopathy	NE	X	NE
Thoracic edema	ANA	X	ANA
Malignant pustule edema	ANA	X	ANA
**Neurologic**			
Altered mental status	X	ANA	ANA
Meningeal signs	X	NE	NE
Nonheadache, nonmeningeal neurologic signs	X	X	NE
**Diagnostic laboratory test result**			
Bacteremia at presentation	ANA	ANA	X
Bacteremia at any time throughout hospitalization	ANA	X	NE

**Abbreviations:** ANA = analyzed and not associated; NE = not evaluated; X = predictive of meningitis.

* **Source:** Binney S, Person MK, Traxler RM, Cook R, Bower WA, Hendricks K. Algorithms for the identification of anthrax meningitis during a mass casualty event based on a systematic review of systemic anthrax from 1880 through 2018. Clin Infect Dis 2022;75(Suppl 3):S468–77.

^†^ Results from Binney et al. and Thompson et al. shared overlapping data sets.

^§^ **Source:** Thompson JM, Cook R, Person MK, et al. Risk factors for death or meningitis in adults hospitalized for cutaneous anthrax, 1950–2018: a systematic review. Clin Infect Dis 2022;75(Suppl 3):S459–67.

^¶^ **Source:** Kutmanova A, Zholdoshev S, Roguski KM, et al. Risk factors for severe cutaneous anthrax in a retrospective case series and use of a clinical algorithm to identify likely meningitis and evaluate treatment outcomes, Kyrgyz Republic, 2005–2012. This patient population included one patient aged <18 years with different cutoffs for abnormal vital signs (i.e., >38.5°C for fever, >110 beats per minute for tachycardia, and >14 breaths per minute for tachypnea).

#### Triage Tool for Predicting Meningitis in a Mass Casualty Event

 In 2016, a study described a 4-item anthrax meningitis assessment (i.e., screening) tool that included severe headache, altered mental status, meningeal signs, and other neurologic signs (*30*). Using this tool, patients were deemed likely to have meningitis if two or more of the items were present and unlikely to have meningitis if no items were present. Patients with only one of the items were in a middle group that would need diagnostic testing (e.g., imaging and lumbar puncture) to determine their meningitis status.

A study (*41*) validated the meningitis screening tool (*30*) and another evaluated newly identified risk factors (*40*). On re-evaluation, the 2016 screening tool had a sensitivity of 86% (95% CI = 71%–100%) and a specificity of 99% (95% CI = 97%–100%). However, >17% of adults still needed further diagnostic workup to determine presence or absence of meningitis. A new, more complex screening tool was developed to minimize the number of patients who would require further testing (Figure). This screening tool had a sensitivity of 86% (95% CI = 71%–100%) and a specificity of 92% (95% CI = 85%–99%) but left only 2.5% of adults in need of further testing (*41*).

**FIGURE:**
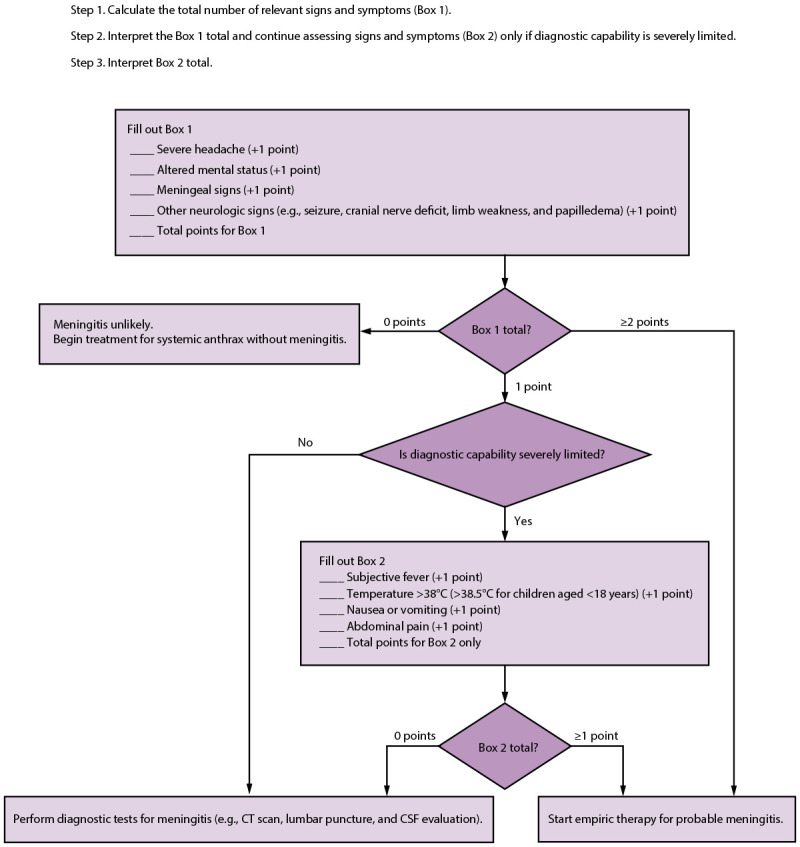
Screening tool to identify potential anthrax meningitis cases by presenting signs and symptoms after a mass casualty event when diagnostic capability is limited — CDC, 2023 **Abbreviations:** CSF = cerebrospinal fluid; CT = computed tomography.

**Source:** Adapted from Figure 2 in Binney S, Person MK, Traxler RM, Cook R, Bower WA, Hendricks K. Algorithms for the identification of anthrax meningitis during a mass casualty event based on a systematic review of systemic anthrax from 1880 through 2018. Clin Infect Dis 2022;75(Suppl 3):S468–77.

#### Antimicrobial Drug Selection

 In the Kyrgyz Republic case series, 37 patients were categorized by an algorithm as likely having meningitis and 89 were considered unlikely to have meningitis (*52*). Twenty-three (62%) of the 37 received monotherapy, and all 23 survived. The remainder (38%) received 2-drug combination therapy and survived. Only five of 14 survivors who received combination therapy received a bactericidal and a PSI antimicrobial drug, and none received three concurrent antimicrobial drugs.

In the systematic review of human treatment data, survival was not improved by adding a PSI to a bactericidal antimicrobial drug in adults with anthrax meningitis (*39*). However, median length of stay for the three patients who received both a PSI and a bactericidal antimicrobial drug was 10 days (IQR = 8–21 days) compared with 30 days (IQR = 15–50 days) for the four who received bactericidal antimicrobial drugs alone, although this difference was not statistically significant (p = 0.26) (*39*).

#### Population PK Modeling to Predict Drug Exposures in Cerebrospinal Fluid for Naturally Occurring *B. anthracis* Strains

 Antimicrobial drugs with published human cerebrospinal fluid (CSF) penetration data were categorized according to their ability to achieve robust drug exposures in both plasma and CSF based on population PK modeling followed by Monte Carlo simulations. Because PK/PD target values for *B. anthracis* are scarce, established targets from other pathogens were used to evaluate drug exposures. One study suggested the PK/PD targets for the carbapenem class of antibiotics (e.g., faropenem) were comparable between *B. anthracis* and gram-negative pathogens. The blood-brain barrier can be impaired considerably during anthrax meningitis; therefore, both the plasma and the CSF exposures were considered. Because of the need for between-pathogen extrapolations of PK/PD targets, absence of CSF-specific targets, and relative sparsity of the data, these Monte Carlo simulation results should be interpreted conservatively.

The Monte Carlo simulations indicated the probability that patients with suspected or documented anthrax meningitis would achieve microbiologic cure of CSF when treated with the recommended dosage was high (≥95%) for ampicillin, ampicillin/sulbactam, ciprofloxacin, doxycycline, imipenem/cilastatin, levofloxacin 500 mg every 12 hours, meropenem, minocycline, and penicillin G; acceptable (≥90% to <95%) for levofloxacin 750 mg every 24 hours and piperacillin/tazobactam; and low (˂90%) for clindamycin, linezolid, and vancomycin (Table 5). Despite poor coverage for bacterial killing, the reduced toxin production provided by PSIs (e.g., clindamycin and linezolid) might still be of benefit and should be part of combination therapy.

**TABLE 5:** Monte Carlo simulations to predict probability of microbiologic treatment success for suspected or documented anthrax meningitis for antimicrobial drugs at specific intravenous dosages

Antimicrobial drug	Drug class	Mechanism of action	IV antimicrobial drug dosage for 70-kg adult	MIC_90_ (*µ*g/mL)	PK/PD breakpoint (*µ*g/mL)*^,†^
**High probability^§^**					
Ciprofloxacin	Fluoroquinolone	C	400 mg every 8 hours	0.03	0.06
Levofloxacin	Fluoroquinolone	C	500 mg every 12 hours	0.25	0.25^¶^
Meropenem	Carbapenem	C	2 g every 8 hours in a 30-minute infusion	0.12	0.25**
Imipenem/cilastatin	Carbapenem/dehydropeptidase inhibitor	C	1 g every 6 hours in a 1-hour infusion	0.12	1**
Penicillin G	Penicillin	C	4 million units every 4 hours	0.03^††^	0.25**
Ampicillin	Penicillin	C	2 g every 4 hours	0.03^††^	0.5**
Ampicillin/sulbactam	Penicillin/ß-lactamase inhibitor	C	3 g every 6 hours	0.03^††^	0.25**
Doxycycline	Tetracycline	S	100 mg every 12 hours	0.03	0.25^¶^
Minocycline	Tetracycline	S	100 mg every 12 hours	≤0.06	0.125^¶^
**Acceptable probability^§§^**					
Piperacillin/tazobactam	Penicillin/ß-lactamase inhibitor	C	3.375 g every 4 hours	1^††^	1
Levofloxacin	Fluoroquinolone	C	750 mg every 24 hours	0.25	0.125^¶^
**Low probability^¶¶^**					
Vancomycin	Glycopeptide	C	15 mg/kg every 12 hours	2	0.06***
Amikacin	Aminoglycoside	C	25 mg/kg every 24 hours	1	0.125**
Clindamycin^†††^	Lincosamide	S	900 mg every 8 hours	0.25	0.0313^§§§^
Linezolid^†††^	Oxazolidinone	S	600 mg every 12 hours	2	0.25***

**Source:** CDC unpublished study.

**Abbreviations:** C = bactericidal; CSF = cerebrospinal fluid; fAUC/MIC = area under the unbound (i.e. free) concentration time curve divided by the minimal inhibitory concentration; fT>MIC = duration of the unbound (i.e. free) concentration above the minimum inhibitory concentration (expressed as a fraction of the dosing interval); IV = intravenous; MIC = minimum inhibitory concentration; MIC_90_ = minimum inhibitory concentration required to inhibit the growth of 90% of organisms; PD = pharmacodynamic; PK = pharmacokinetic; S = bacteriostatic.

* Highest MIC (*µ*g/mL) with a >95% probability of successful CSF treatment.

^†^ PK/PD targets used were fAUC/MIC ≥18 for doxycycline and minocycline, fAUC/MIC ≥75 for quinolones, fT>MIC ≥40% for carbapenems, fT>MIC ≥50% for penicillins, fAUC/MIC ≥90 for amikacin, fAUC/MIC ≥27 for clindamycin, AUC/MIC ≥110 for linezolid, and AUC/MIC ≥400 for vancomycin.

^§^ High probability is defined as a ≥95% probability of treatment success.

^¶^ PK/PD target achievement and the highest treatable MICs were similar (i.e., within twofold) in CSF and plasma for ciprofloxacin, levofloxacin, doxycycline, and minocycline.

** For the studied ß-lactams (i.e., meropenem, imipenem, penicillin G, ampicillin, and piperacillin) and for amikacin, the highest treatable MICs based on the plasma targets were twofold to thirty-two-fold higher compared with those in CSF.

^††^ Previous susceptibility testing to infecting strain is recommended. *Bacillus anthracis* naturally carries penicillin-resistance genes for a ß-lactamase that is expressed in up to 10% of naturally occurring strains, creating resistance to penicillin G, ampicillin, and piperacillin.

^§§^ Acceptable probability is defined as a ≥90% to <95% probability of treatment success; these antimicrobial drugs should be used only if those that provide high probability of success are not available.

^¶¶^ Low probability is defined as a ˂90% probability of treatment success.

*** For linezolid and vancomycin, the highest treatable MICs based on plasma drug exposures were fourfold to eightfold below the MIC_90_.

^†††^ Clindamycin and linezolid did not achieve sufficient drug exposures in CSF and plasma as monotherapy to achieve clinically relevant bacterial killing. However, these protein synthesis inhibitors might be useful in combination therapy to inhibit toxin production.

^§§§^ For clindamycin, the highest treatable MIC based on plasma drug exposures was 0.25 *µ*g/mL (MIC_90_).

#### Intracerebral Hemorrhage With Anthrax Meningitis

Anthrax meningitis is often accompanied by destruction of the blood-brain barrier and intracranial bleeding and swelling (*51*). Subarachnoid hemorrhage is a common complication (*51*). Meningeal seeding and a metalloprotease of *B. anthracis* that targets the tight junctions of the blood-brain barrier contribute to destruction of the barrier and cerebral hemorrhage. Both animal experimental models and case reports suggest that intracranial hemorrhage is common when *B. anthracis* infection involves the brain (*65*,*66*). The infection combined with neurotoxicity of intracranial blood likely leads to rapid brain swelling and the poor outcomes observed among these patients (*67*).

Recent progress has been made in the treatment of aneurysmal subarachnoid and spontaneous intracerebral hemorrhage. Nimodipine is the only drug approved by FDA for aneurysmal subarachnoid hemorrhage and reduces the incidence of poor neurologic outcome by 40% (*68*). In vivo studies also have suggested nimodipine might be beneficial in treating pneumococcal meningitis (*69*,*70*). Although these principles of neurointensive care and treatment regimens are used routinely in patients with bleeds, they appear to have seldom been applied in patients with anthrax meningitis. Patients with anthrax meningitis might benefit from standard treatment for intracerebral hemorrhage.

Certain antimicrobial drugs demonstrated to have neuroprotective effects in other diseases or models might be useful for anthrax meningitis. For example, minocycline is a highly lipophilic second-generation tetracycline that readily crosses the blood-brain barrier and has been observed in vivo to have neuroprotective effects against subarachnoid hemorrhage (*71*–*75*), intracerebral bleeding (*76*–*81*), and blood-brain barrier disruption (*71*,*74*–*76*,*81*–*89*). Theoretically, minocycline might be beneficial for the treatment of anthrax meningitis because of its anti-inflammatory, antiapoptotic, and antioxidant effects (*90*–*92*). Other antimicrobial drugs with in vivo neuroprotective effects for certain meningitides include ß-lactams (*93*), clindamycin (*94*), and daptomycin (*95*,*96*).

#### Cerebral Edema in Anthrax Meningitis

In adult patients with systemic anthrax, the odds of survival for the steroid and no steroid groups did not statistically differ after adjusting for age and severity (*39*). For adults with meningitis, survival was not different in those who received steroids (two of six) compared with those who did not (three of 39; OR = 6.00; 95% CI = 0.76–47.36). However, patients who received mannitol had higher odds of survival than those who did not (OR = 24.00; 95% CI = 1.66–347.85). For example, two of the rare survivors of anthrax meningitis received mannitol or mannitol with hyperosmolar therapy (*49*,*97*). In addition, bacterial meningitis studies in animal models found that 3% hypertonic saline reduces intracranial pressure, improves cerebral perfusion pressure, inhibits aquaporin-4 expression, reduces cerebral edema, and attenuates neuronal injury. This suggests hypertonic saline might be beneficial for the treatment of symptomatic cerebral edema (*98*).

### Review of Safety Data for Antimicrobial Drugs and Antitoxins Proposed for PEPAbx and Treatment of Anthrax

When evaluating the benefit of antimicrobial drugs for PEPAbx or treatment of anthrax, or both, risks common to antimicrobial drugs (e.g., hypersensitivity, *C.* *difficile* infection and associated diarrhea, and selective pressure for colonization and subsequent infection by resistant organisms) should be considered in the risk-benefit evaluation. Additional considerations should include specific adverse events for each antimicrobial drug and antimicrobial drug class and patient- and population-specific characteristics that influence antimicrobial drug selection (e.g., renal function, drug allergies, and interacting concomitant medications). The recommended antimicrobial drugs have known or potential serious or severe risks that might influence their selection and prioritization for PEPAbx or treatment of anthrax across different patient populations. The safety review that evaluated 24 selected antimicrobials under consideration for PEPAbx or treatment of anthrax, or both, concluded that, for the reviewed antimicrobial drugs, because of the considerable morbidity and mortality associated with anthrax, the risk-benefit evaluation favors their consideration for anthrax, provided risk-mitigation measures are implemented as warranted (*40*).

#### Fluoroquinolones

Warnings for all fluoroquinolones include risk for central nervous system effects (e.g., serious psychotic reactions), tendon rupture and tendonitis, peripheral neuropathy, aortic aneurysm and dissection, hepatotoxicity, and altered cardiac conduction. Older persons might be at increased risk for these AEs. Although fluoroquinolones generally are not recommended for routine first-line use in pregnant persons and children, multiple studies found that the fluoroquinolones were well tolerated in the pediatric population (*99*–*102*). In addition, an American Academy of Pediatrics report supports use of fluoroquinolones in children when clinically necessary (*103*).

#### Tetracyclines

 Historically, FDA labeling for tetracycline-class antimicrobial drugs has indicated that they should only be used in children aged <8 years for severe conditions when the benefits of treatment are likely to exceed the risks. More recent literature suggests that tooth staining is uncommon with doxycycline; thus, up to 21 days of doxycycline use is now acceptable for all children with conditions for which tetracyclines are the preferred class (*104*,*105*).

##### Penicillins

The penicillin class is generally well tolerated in all populations. A previous FDA review supported the safety of long-term penicillin and penicillin-derivative use (*106*).

#### Carbapenems

 Serious AEs associated with the carbapenem class (especially imipenem/cilastatin) include seizure. For this reason, imipenem/cilastatin is not advised for treatment of infections involving the central nervous system (*107*,*108*). If treatment with imipenem/cilastatin is needed for anthrax meningitis, antiepileptic agents can be added to reduce seizure risk, including in the pediatric population (*109*).

#### Other Antimicrobial Drugs

 The prolonged use of clindamycin for PEP might increase the risk for *C. difficile* infection and associated diarrhea (*110*–*112*). The prolonged use of linezolid for PEP might increase the risk for myelosuppression, lactic acidosis, and potentially irreversible optic neuritis and peripheral neuropathy (*113*,*114*).

#### Anthrax Antitoxins

 AIGIV can cause thrombosis and false high readings with certain point-of-care blood glucose testing systems (*115*). Both obiltoxaximab and raxibacumab can cause hypersensitivity reactions and anaphylaxis (*116*–*118*). Patients receiving either monoclonal antitoxin should be premedicated with diphenhydramine.

## CDC Recommendations for Prevention and Treatment of Anthrax

Anthrax can be a devastating disease. In one Russian series, one in six patients with cutaneous or ingestion exposures to *B. anthracis* developed anthrax (*62*). Mortality rates for adequately treated anthrax range from <2% (*119*) for cutaneous anthrax to 45% for inhalation anthrax (*13*,*120*) and >90% for anthrax meningitis (*29*,*52*). On the basis of efficacy described from in vivo data and human clinical experience and known potential risks, the benefits of antimicrobial drugs for PEP or treatment of anthrax outweigh the known risks.

These guidelines address anthrax PEP and treatment for both natural and intentional exposures (e.g., a wide-area aerosol release of *B. anthracis* spores). The evidence base linking recommendations to data is available (Supplementary Material, https://stacks.cdc.gov/view/cdc/132182). Previously, all *B. anthracis* strains from a naturally occurring source or an intentional release were thought to be susceptible to the recommended first-line antimicrobial drugs (except for penicillins). However, over the past few decades, studies have demonstrated that antimicrobial-resistant *B. anthracis* strains can be created with relative ease through serial passaging on selective media (*121*,*122*). Consequently, bioterrorists could mass produce a multidrug-resistant *B. anthracis* strain capable of evading previously recommended first-line antimicrobial medical countermeasures. These updated CDC guidelines provide PEP and treatment recommendations that include numerous antimicrobial drugs from multiple classes. The antimicrobial drugs recommended as first-line agents are expected to address most scenarios. The alternative antimicrobial drugs provide contingencies for contraindications, intolerances, unavailability, and natural or genetically engineered resistance.

The recommended medical countermeasures are preferentially ordered based on 1) in vitro effectiveness against *B. anthracis* (*34*) (Table 1); 2) in vivo efficacy against *B. anthracis* exposures as demonstrated by ORs and CIs for survival compared with no therapy or therapy with a positive control (*35*,*37*) (Table 2); 3) the animal model used to generate efficacy data (nonhuman primate or rabbit models were preferred over mouse, guinea pig, or hamster models); 4) treatment outcomes for published human cases (*39*) (Table 3); 5) the percentage of patients expected to achieve microbiologic CSF cure at recommended antimicrobial drug dosing based on Monte Carlo simulations (Table 5); 6) the safety profiles of the antimicrobial drugs (*42*); 7) logistical considerations (e.g., available formulations [including availability and palatability of liquid formulations], dosing intervals, cost, and supply and availability patterns); and 8) expert opinion. In addition, certain antimicrobial drugs are included for PEPAbx or treatment on the basis of class efficacy (e.g., levofloxacin, moxifloxacin, and ofloxacin) or for treatment on the basis of demonstrated PEPAbx efficacy (e.g., minocycline).

Early diagnosis of anthrax and initiation of appropriate treatment are critical to improving survival. Although empiric treatment of anthrax or prophylaxis after exposure is needed to save lives, antimicrobial drug susceptibility testing is vital; antimicrobial drug choices might need to be modified based on the results. Data indicate penicillin-class antimicrobial drugs are as effective as other bactericidal agents for PEPAbx and treatment and might be preferred in certain populations. However, although <10% of naturally occurring *B. anthracis* isolates are reported to be resistant to penicillin-class antimicrobial drugs (*123*–*126*), these drugs should only be used if the strain is known to be penicillin susceptible. In vitro data demonstrate that cephalosporins, trimethoprim/sulfamethoxazole, and aztreonam are ineffective against *B. anthracis*. If liquid formulations are not available for children or adults who cannot swallow pills, instructions are available for preparing oral suspensions of moxifloxacin (*127*) and doxycycline (*128*).

### Nonpregnant Adults Aged ≥18 Years

#### PEP and Treatment Regimens for Cutaneous Anthrax Without Signs and Symptoms of Meningitis

PEP regimens for nonpregnant adults aged ≥18 years exposed to *B. anthracis* include either a single antimicrobial drug or, if antimicrobial drugs are not available, a single anthrax antitoxin (Table 6). Both antimicrobial drugs and antitoxins are highly effective at preventing disease in animals. However, because antitoxins are administered intravenously and are somewhat (i.e., the monoclonals) to moderately (i.e., the polyclonal) less efficacious than antimicrobial drugs (*35*,*37*), all oral antimicrobial drugs are preferred over antitoxins. In addition, in a wide-area aerosol release of *B. anthracis* spores, antitoxins should be prioritized for treatment rather than PEP because they likely provide greater benefit as adjunctive treatments. If coadministration of anthrax vaccine and antitoxin is indicated, the only antitoxin that should be used is raxibacumab (*37*).

**TABLE 6:** Empiric* postexposure prophylaxis for nonpregnant adults aged ≥18 years after exposure to *Bacillus anthracis*, by descending order of preference — CDC recommendations, 2023

Treatment (listed drugs joined by “or” are considered equivalent)	Dosage
**First-line antimicrobial drug**	
Doxycycline^†,§^	100 mg every 12 hours orally
or	
Ciprofloxacin^†^	500 mg every 12 hours orally
or	
Levofloxacin^†^	500 mg every 24 hours orally
PCN-S only:	
Amoxicillin^¶,^**	1 g every 8 hours orally
Penicillin VK^¶^	500 mg every 6 hours orally
**Alternative antimicrobial drug^††^**	
Minocycline^†^	200 mg x 1 dose orally, then 100 mg every 12 hours orally
Amoxicillin/clavulanate^¶^	16:1 formulation (1 g/62.5 mg) in 2 tablets every 12 hours orally
or	
Amoxicillin/clavulanate^¶^	7:1 formulation (875/125 mg) every 12 hours orally
Moxifloxacin^§,¶^	400 mg every 24 hours orally
Ofloxacin^¶^	400 mg every 12 hours orally
Clindamycin^¶^	600 mg every 8 hours orally
Omadacycline^¶^	450 mg every 24 hours orally x 2 days, then 300 mg every 24 hours orally
Linezolid^¶,§§^	600 mg every 12 hours orally
Tetracycline^†^	500 mg every 6 hours orally
Clarithromycin^¶,¶¶^	500 mg every 12 hours orally (only initiate after at least 3 days of treatment with any of the other antimicrobial drugs listed)
Dalbavancin^¶^	1 g x 1 dose IV, then 500 mg weekly IV
**Antitoxin (only to be used as PEP if antimicrobial drugs are not available or not appropriate; listed antitoxins joined by “or” are considered equivalent)**	
Raxibacumab***	40 mg/kg as a single dose IV
or	
Obiltoxaximab***	16 mg/kg as a single dose IV

**Abbreviations:** FDA = Food and Drug Administration; PCN-S = penicillin-susceptible strains: PEP = postexposure prophylaxis; PEPAbx = antimicrobial postexposure prophylaxis for anthrax.

* Definitive therapy should be directed by antibiotic susceptibility test results, when available.

^†^ Approved by FDA for anthrax PEPAbx, treatment, or both, but specific uses (e.g., doses, dosing schedules, and patient populations) recommended in this report might differ from the FDA-approved labeling.

^§^ If liquid formulations are not available for adults who cannot swallow pills, instructions are available for preparing oral suspensions of moxifloxacin (**Source:** Hutchinson DJ, Johnson CE, Klein KC. Stability of extemporaneously prepared moxifloxacin oral suspensions. Am J Health Syst Pharm 2009;66:665–7.121) and doxycycline (**Source:** CDC. In an anthrax emergency: how to prepare doxycycline hyclate for children and adults who cannot swallow pills. Atlanta, GA: US Department of Health and Human Services, CDC; 2020. https://www.cdc.gov/anthrax/public-health/doxy-crushing-instruction-pamphlet.html).

^¶^ Not approved by FDA for anthrax PEPAbx or treatment.

** Ampicillin 500 mg every 6 hours orally can be used as an alternative to amoxicillin, if available.

^††^ Alternative selections are for patients who have contraindications to or cannot tolerate first-line antimicrobial drugs or if first-line antimicrobial drugs are not available.

^§§^ Linezolid can be considered for PEP in scenarios when patients can receive regular monitoring for myelosuppression or neurotoxicity, which might occur within 14–28 days of use. If possible, switch to an alternative drug when available.

^¶¶^ Clarithromycin is unlikely to be effective if the patient has bacteremia, thus a different antimicrobial drug must be used initially to clear bacteremia.

*** Premedicate with IV or oral diphenhydramine within 1 hour before administration. Hypersensitivity and anaphylaxis have been reported after raxibacumab and obiltoxaximab administration.

Studies in animal models (*129*,*130*) and a report after an accidental wide-area aerosol release of *B. anthracis* spores (*15*) suggest the incubation period for inhalation anthrax in those administered PEPAbx might be up to 60 days. To prevent anthrax after discontinuation of PEPAbx, ACIP recommends AVA for adults aged 18–65 years in conjunction with a course of PEPAbx (*33*). AVA is administered subcutaneously at 0, 2, and 4 weeks postexposure; it can be administered intramuscularly if the subcutaneous route poses significant materiel, personnel, or clinical challenges. AVA can be used under an appropriate regulatory mechanism (e.g., investigational new drug or emergency use authorization) in persons aged <18 years and >65 years exposed to anthrax. In July 2023, a second-generation anthrax vaccine, anthrax vaccine adsorbed, adjuvanted, was FDA approved for PEPVx against inhalation anthrax. Anthrax vaccine adsorbed, adjuvanted is administered by the IM route as a 2-dose series 2 weeks apart, in conjunction with PEPAbx for adults aged 18–65 years. In persons aged >65 years, anthrax vaccine adsorbed, adjuvanted elicited a higher immune response compared with AVA (*131*). Anthrax vaccine use in older adults (aged >65 years), pregnant or lactating persons, and children (aged <18 years) would be guided by data available at the time of an anthrax event.

For nonpregnant adults aged ≥18 years, antimicrobial drug monotherapy can be used for treatment of both localized and systemic cutaneous anthrax if the patient does not have signs and symptoms of meningitis (*37*) (Table 7). A penicillin-class antimicrobial drug can be used as monotherapy if the organism is known to be penicillin susceptible, which will allow combination regimens to be reserved for patients with high-mortality forms of anthrax (e.g., inhalation anthrax). Anthrax antitoxin can be used to treat cutaneous anthrax without signs and symptoms of meningitis if all recommended antimicrobial drugs are not available or not appropriate.

**TABLE 7:** Empiric* treatment regimens for nonpregnant adults aged ≥18 years with cutaneous anthrax without signs and symptoms of meningitis, by descending order of preference — CDC recommendations, 2023

Treatment (listed drugs joined by “or” are considered equivalent)	Dosage
**First-line antimicrobial drug**	
Doxycycline^†,§^	100 mg every 12 hours orally
or	
Minocycline^†^	200 mg x 1 dose orally, then 100 mg every 12 hours orally
or	
Ciprofloxacin^†^	500 mg every 12 hours orally
or	
Levofloxacin^†^	750 mg every 24 hours orally
PCN-S only:	
Amoxicillin^¶,^**	1 g every 8 hours orally
or	
Penicillin VK^¶^	500 mg every 6 hours orally
**Alternative antimicrobial drug^††^**	
Amoxicillin/clavulanate^¶^	1:16 formulation (1 g/62.5 mg) in 2 tablets every 12 hours orally
or	
Amoxicillin/clavulanate^¶^	1:7 formulation (875/125 mg) every 12 hours orally
Moxifloxacin^§,¶^	400 mg every 24 hours orally
Clindamycin^¶^	600 mg every 8 hours orally
Ofloxacin^¶^	400 mg every 12 hours orally
Omadacycline^¶^	450 mg every 24 hours orally x 2 days, then 300 mg every 24 hours orally
Linezolid^¶^	600 mg every 12 hours orally
Tetracycline^†^	500 mg every 6 hours orally
Clarithromycin^¶,§§^	500 mg every 12 hours orally (only initiate after at least 3 days of treatment with any of the other antimicrobials listed)
Dalbavancin^¶^	1 g x 1 dose IV, then 500 mg weekly IV
Imipenem/cilastatin^¶^	1 g every 6 hours IV
or	
Meropenem^¶^	2 g every 8 hours IV
Vancomycin^¶^	15 mg/kg every 12 hours IV over a period of 1–2 hours (target AUC_24_ of 400–600 *µ*g x h/mL [preferred]; if AUC_24_ is not available, maintain serum trough concentrations of 15–20 *µ*g/mL)
**Antitoxin (only to be used if antimicrobial drugs are not available or not appropriate; listed antitoxins joined by “or” are considered equivalent)**	
Raxibacumab^¶¶^	40 mg/kg in a single dose IV
or	
Obiltoxaximab^¶¶^	16 mg/kg in a single dose IV
AIGIV***	420 units IV

**Abbreviation:** AIGIV = anthrax immunoglobulin intravenous; FDA = Food and Drug Administration; IV = intravenous; PCN-S = penicillin-susceptible strains; PEPAbx = antimicrobial postexposure prophylaxis for anthrax.

* Definitive therapy should be directed by antibiotic susceptibility test results, when available.

^†^ Approved by FDA for anthrax PEPAbx, treatment, or both, but specific uses (e.g., doses, dosing schedules, and patient populations) recommended in this report might differ from the FDA-approved labeling.

^§^ If liquid formulations are not available for adults who cannot swallow pills, instructions are available for preparing oral suspensions of moxifloxacin (**Source:** Hutchinson DJ, Johnson CE, Klein KC. Stability of extemporaneously prepared moxifloxacin oral suspensions. Am J Health Syst Pharm 2009;66:665–7.121) and doxycycline (**Source:** CDC. In an anthrax emergency: how to prepare doxycycline hyclate for children and adults who cannot swallow pills. Atlanta, GA: US Department of Health and Human Services, CDC; 2020. https://www.cdc.gov/anthrax/public-health/doxy-crushing-instruction-pamphlet.html).

^¶^ Not approved by FDA for anthrax PEPAbx or treatment.

** Ampicillin 500 mg every 6 hours can be used as an alternative to amoxicillin, if available.

^††^ Alternative selections are for patients who have contraindications to or cannot tolerate first-line antimicrobial drugs or if first-line antimicrobial drugs are not available.

^§§^ Clarithromycin is unlikely to be effective if the patient has bacteremia, thus a different antimicrobial drug must be used initially to clear bacteremia.

^¶¶^ Premedicate with IV or oral diphenhydramine within 1 hour before administration. Hypersensitivity and anaphylaxis have been reported after raxibacumab and obiltoxaximab administration.

*** An 840-unit dose of AIGIV can be considered for severe cases.

For nonpregnant adults aged ≥18 years, empiric PEP (Table 6) and empiric cutaneous anthrax treatment (Table 7) regimens include either a single antimicrobial drug or a single antitoxin and are summarized as follows:

- Antimicrobial drug: Choose a single antimicrobial drug.
  º Antimicrobial drugs are listed in descending order of preference in the table. Listed drugs joined by “or” are considered equivalent.
  ° Continue or switch antimicrobial drug based on susceptibility testing once available.
  ° Only choose a “PCN-S only” antimicrobial drug after the strain has been determined to be penicillin susceptible.
- Antitoxin: Choose a single antitoxin if no antimicrobial drugs are available.

For adults aged 18–65 years, when PEPAbx is used without PEPVx after aerosol exposure (e.g., a bioterrorism-related incident or animal skin drum–related event), PEPAbx should be continued for 60 days. When PEPAbx is used with PEPVx for healthy, nonpregnant adults aged 18–65 years, antimicrobial drugs can be discontinued 42 days after the first dose or 2 weeks after the last dose of vaccine, whichever occurs later. For older adults (aged ≥66 years) and persons with immunocompromising conditions, PEPAbx should continue for 60 days (*33*). For adults aged 18–65 years with nonaerosol (i.e., cutaneous or ingestion) exposures, PEPAbx should continue for 7 days and vaccine is not needed.

For adults aged 18–65 years with cutaneous anthrax without signs and symptoms of meningitis, the treatment regimen should continue for 7–10 days, or until clinical criteria for stability are met. If an aerosol exposure might have occurred, patients should transition from a treatment to a PEP regimen (Table 6); the combined regimen should total 42–60 days from exposure, depending on anthrax vaccine status and immunocompetence. If no aerosolizing event occurred, patients with cutaneous anthrax do not need to continue PEPAbx.

#### Treatment Regimens for Systemic Anthrax With or Without Meningitis

For nonpregnant adults aged ≥18 years with systemic anthrax with or without meningitis, bactericidal agents have been found to provide a survival benefit compared with other agents (*37*) and are preferred over PSIs (Table 8). In vivo and observational clinical data for systemic anthrax have not demonstrated a survival benefit for combination antimicrobial drug therapy over monotherapy. However, translating these analyses to patient treatment is challenging because various animal models and nonvirulent *B. anthracis* strains were used; the clinical data were retrospective, observational, and drawn from medical literature that is subject to reporting bias; and only a limited number of patients belonged to a particular treatment category.

**TABLE 8:** Empiric* treatment regimens for nonpregnant adults aged ≥18 years with systemic^†^ anthrax with or without meningitis,^§^ by descending order of preference — CDC recommendations, 2023

Regimen		Example	
Regimen 1. Two bactericidal drugs from different antimicrobial drug classes plus a PSI or an RNAI		Ciprofloxacin plus meropenem plus minocycline^¶^	
Regimen 2. One bactericidal drug plus a PSI		Meropenem plus doxycycline	
Regimen 3. One bactericidal drug plus a second bactericidal drug from a different antimicrobial drug class		Meropenem plus ciprofloxacin	
Regimen 4. One bactericidal drug plus an RNAI (rifampin should not be used as monotherapy)		Meropenem plus rifampin	
Regimen 5. A PSI plus an RNAI (rifampin should not be used as monotherapy)		Minocycline or doxycycline plus rifampin	
Regimen 6. Two PSIs from different antimicrobial drug classes		Minocycline plus clindamycin	
Regimen 7. A single bactericidal drug		Meropenem	
Regimen 8. A single PSI		Minocycline or doxycycline or clindamycin	
First-line antimicrobial drug**			
**Bactericidal drug**		**PSI**	
**Treatment (listed drugs joined by “or” are considered equivalent)**	**Dosage**	**Treatment**	**Dosage**
Meropenem^††^	2 g every 8 hours IV	Minocycline^§§^	200 mg x 1 dose IV, then 100 mg every 12 hours IV
or			
Ciprofloxacin^§§^	400 mg every 8 hours IV	Doxycycline^§§^	200 mg x 1 dose IV, then 100 mg every 12 hours IV
or			
Levofloxacin^§§^	500 mg every 12 hours IV		
PCN-S only:			
Penicillin G^§§^	4 million units every 4 hours IV		
or			
Ampicillin^††^	2 g every 4 hours IV		
Imipenem/cilastatin^††^	1 g every 6 hours IV		
or			
Ampicillin/sulbactam^††^	3 g every 6 hours IV		
**Alternative antimicrobial drug^¶¶^**			
**Bactericidal drug**		**PSI/RNAI**	
**Treatment**	**Dosage**	**Treatment**	**Dosage**
Piperacillin/tazobactam^††^	3.375 g every 4 hours IV	Omadacycline^††,^***	200 mg x 1 dose IV on day 1, then 100 mg every 24 hours IV
Moxifloxacin^††^	400 mg every 24 hours IV	Eravacycline^††,^***	1 mg/kg every 12 hours IV
Vancomycin^††,^***	15 mg/kg every 12 hours IV over a period of 1–2 hours (target AUC_24_ of 400 *µ*g x h/mL [preferred]; if AUC_24_ is not available, maintain serum trough concentrations of 15–20 *µ*g/mL). Consider a loading dose of 20–35 mg/kg for critically ill patients.	Clindamycin^††^	900 mg every 8 hours IV
		Linezolid^††^	600 mg every 12 hours IV
		Rifampin^††,†††^	600 mg every 12 hours IV
		Chloramphenicol^††,§§§^	1 g every 6–8 hours IV
plus			
**Antitoxin (single dose as an adjunct to antimicrobial drug; listed antitoxins joined by “or” are considered equivalent)**			
**Treatment**		**Dosage**	
Raxibacumab^¶¶¶^		40 mg/kg IV	
or			
Obiltoxaximab^¶¶¶^		16 mg/kg IV	
AIGIV****		420 units IV	

**Abbreviations:** AIGIV = anthrax immunoglobulin intravenous; AUC_24_ = area under the concentration-time curve from 0 to 24 hours; FDA = Food and Drug Administration; IV = intravenous; PCN-S = penicillin-susceptible strains; PEPAbx = antimicrobial postexposure prophylaxis for anthrax; PSI = protein synthesis inhibitor; RNAI = RNA synthesis inhibitor.

* Definitive therapy should be directed by antibiotic susceptibility test results, when available.

^†^ “Systemic” was defined as one or more of the following using cutoffs for adults aged ≥18 years: hyperthermia or hypothermia, tachycardia, tachypnea, hypotension, or neutrophilia or neutropenia (**Source:** Katharios-Lanwermeyer S, Holty JE, Person M, et al. Identifying meningitis during an anthrax mass casualty incident: systematic review of systemic anthrax since 1880. Clin Infect Dis 2016;62:1537–45).

^§^ Refer to Figure for guidance on clinical signs and symptoms of anthrax meningitis. If meningitis is not suspected and susceptibilities are known, start at regimen 2.

^¶^ For anthrax meningitis, consider using antimicrobial drugs that have demonstrated potential neuroprotective benefits in vivo (e.g., minocycline, doxycycline, clindamycin, and ß-lactamase inhibitors).

** For highly bioavailable antimicrobial drugs (e.g., ciprofloxacin, doxycycline, and linezolid), if the IV formulation is not available, oral formulations can be considered for patients with an intact gastrointestinal tract where absorption is expected to be complete after oral administration.

^††^ Not approved by FDA for anthrax PEPAbx, treatment, or both.

^§§^ Approved by FDA for anthrax PEPAbx, treatment, or both, but specific uses (e.g., doses, dosing schedules, and patient populations) recommended in this report might differ from the FDA-approved labeling.

^¶¶^ Alternative selections are for patients who have contraindications to or cannot tolerate first-line antimicrobial drugs or if first-line antimicrobial drugs are not available.

*** This antimicrobial drug does not cross an intact blood-brain barrier but might cross with meningitis because of breakdown of the barrier.

^†††^ Rifampin is an RNAI and also bactericidal; however, it should not be used as monotherapy. Rifampicin is equivalent to rifampin and can be used if it is more readily available.

^§§§^ Chloramphenicol should not be used in combination with a bactericidal antimicrobial drug because the interaction might be antagonistic.

^¶¶¶^ Premedicate with IV or oral diphenhydramine within 1 hour before administration. Hypersensitivity and anaphylaxis have been reported after raxibacumab and obiltoxaximab administration.

**** An 840-unit dose of AIGIV can be considered for severe cases.

In contrast, issues surrounding toxin production support at least initial use of combination therapy. Production of one of the *B. anthracis* virulence toxins (i.e., protective antigen) was found to be reduced in vitro by linezolid (*132*) and both in vitro and in vivo by clindamycin (*133*). Ciprofloxacin plus clindamycin demonstrated survival benefit over ciprofloxacin alone in a rabbit model using a virulent *B. anthracis* strain; the benefit was attributed to inhibition of toxin synthesis by clindamycin (*133*). In a retrospective analysis of inhalation anthrax among patients receiving heterogeneous treatment, patients treated earlier (before fulminant infection) who received combination antimicrobial drug therapy (ciprofloxacin, clindamycin, and rifampin) experienced a survival advantage over those who received a single antimicrobial drug (*28*). Finally, combination therapy with a bactericidal antimicrobial drug and a PSI is recommended to rapidly reduce toxin production for other high-mortality toxin-mediated diseases (e.g., necrotizing fasciitis and streptococcal toxic shock syndrome) (*134*).

The potential for natural and genetically engineered antimicrobial drug–resistant strains also supports at least initial use of combination therapy. Up to 10% of naturally acquired anthrax can be resistant to penicillin-based treatments, and *B. anthracis* strains genetically engineered to be resistant to multiple antimicrobial drugs are an even greater concern (*121*–*126*). Combining two or three antimicrobial drug classes should provide microbiologic activity against most strains that elaborate recognized mechanisms of resistance.

Because of the highly lethal nature of untreated systemic anthrax, particularly when complicated by anthrax meningoencephalitis, combination therapy should be used to address both the toxin-mediated pathogenesis of this infection and potential antibiotic-resistant *B. anthracis*. Empiric treatment regimens for nonpregnant adults aged ≥18 years with systematic anthrax with or without meningitis (Table 8) are summarized as follows:

- Antimicrobial drugs: Choose two bactericidal drugs from different antimicrobial drug classes plus a PSI or an RNA synthesis inhibitor (RNAI).
  º Antimicrobial drugs are listed in descending order of preference in the table. Listed drugs joined by “or” are considered equivalent.
  º Continue or switch antimicrobial drugs based on susceptibility testing once available.
  º Only choose a “PCN-S only” antimicrobial drug after the strain has been determined to be penicillin susceptible.
- Antitoxin: Choose a single antitoxin as adjunctive therapy.

If an appropriate combination of bactericidal antimicrobial drug plus a PSI or an RNAI is contraindicated, not well tolerated, or not available or if meningitis is considered unlikely, consider the following regimens in descending order of preference:

- One bactericidal drug plus a PSI (start with this regimen if meningitis is not suspected and susceptibilities are known)
- One bactericidal drug plus a second bactericidal drug from a different antimicrobial drug class
- One bactericidal drug plus an RNAI
- A PSI plus an RNAI
- Two PSIs from different antimicrobial drug classes
- A single bactericidal drug
- A single PSI

From a PK/PD perspective, minocycline and doxycycline are the preferred PSIs because they provide more robust drug exposures in plasma and CSF compared with clindamycin and linezolid. A single RNAI (i.e., rifampin) should not be used as monotherapy because of the potential for rapid development of resistance (*135*). In addition, when meningitis is not suspected, certain oral formulations are included as alternatives in case IV formulations are not available.

Duration of antimicrobial drug treatment should be 2 weeks or longer; however, duration can be shortened and IV administration transitioned to oral medication based on patient improvement and clinical judgment. Patients with systemic anthrax resulting from a nonaerosolizing event do not need continued antimicrobial drugs for PEP. If an aerosol exposure might have occurred (e.g., a bioterrorism-related incident or animal skin drum–related event), patients treated for systemic disease who are immunocompetent do not need further antimicrobial drugs for PEP because they will have developed natural immunity. However, patients who are immunocompromised should transition to an oral PEP regimen (Table 6). The total duration of antimicrobial drug therapy (i.e., treatment plus PEP) should be 60 days from onset of illness.

#### Antitoxin

 Anthrax antitoxin should be provided as adjunctive therapy to antimicrobial drug regimens for all patients with noncutaneous systemic anthrax. The monoclonal antitoxins are preferred over the polyclonal antitoxin. If antitoxin supplies are likely to be limited, reserving their use for patients developing signs of hemodynamic instability or respiratory compromise is warranted.

### Special Populations

#### Pregnant and Lactating Persons

 A review of historical case reports of anthrax in pregnant and postpartum women found that *B. anthracis* infection in this population is associated with high rates of maternal and fetal death (*136*). The data from the systematic reviews were too sparse to make specific recommendations for pregnant and lactating persons. Thus, the PEPAbx and treatment recommendations for nonpregnant adults aged ≥18 years were the basis for guidelines for pregnant and lactating persons.

Although fluoroquinolones traditionally have not been prescribed during pregnancy and lactation, three recent systematic reviews evaluated their safety during pregnancy. Two systematic reviews (*137*,*138*) found no association between fluoroquinolone exposure throughout pregnancy and adverse pregnancy outcomes, and another found no association between first-trimester fluoroquinolone exposure and adverse pregnancy outcomes (*139*). Tetracycline and minocycline are not recommended in the second and third trimesters of pregnancy because of risk for hepatotoxicity, cardiovascular birth defects, spontaneous abortion, and tooth staining and the potential for transient suppression of bone growth. Data are limited regarding use of eravacycline or omadacycline during pregnancy.

Recommendations for pregnant and lactating persons aged ≥18 years are similar to those for nonpregnant adults except that neither tetracycline nor minocycline are included. This principle applies for empiric PEP (Table 9), empiric treatment of cutaneous anthrax without signs and symptoms of meningitis (Table 10), and empiric treatment of systemic anthrax with or without meningitis (Table 11). A review of doxycycline studies has indicated that doxycycline, unlike other tetracycline-class antimicrobial drugs, has not been associated with fetal growth delays, infant tooth staining, or maternal fatty liver (*140*). Because of the potential severity of anthrax in pregnant and lactating persons, omadacycline and eravacycline can be used if other PSIs are not available.

**TABLE 9:** Empiric* postexposure prophylaxis for pregnant or lactating persons^†,§^ aged ≥18 years after exposure to *Bacillus anthracis*, by descending order of preference — CDC recommendations, 2023

Treatment (listed drugs joined by “or” are considered equivalent)	Dosage
**First-line antimicrobial drug**	
Doxycycline^¶,^**	100 mg every 12 hours orally
or	
Ciprofloxacin^¶^	500 mg every 12 hours orally
or	
Levofloxacin^¶^	500 mg every 24 hours orally
PCN-S only:	
Amoxicillin^††,§§^	1 g every 8 hours orally
Penicillin VK^††^	500 mg every 6 hours orally
**Alternative antimicrobial drug^¶¶^**	
Amoxicillin/clavulanate^††^	16:1 formulation (1 g/62.5 mg) in 2 tables every 12 hours orally
or	
Amoxicillin/clavulanate^††^	7:1 formulation (875/125 mg) every 12 hours orally
Moxifloxacin**^,††^	400 mg every 24 hours orally
Ofloxacin^††^	400 mg every 12 hours orally
Clindamycin^††^	600 mg every 8 hours orally
Omadacycline^††^	450 mg every 24 hours orally x 2 days, then 300 mg every 24 hours orally
Linezolid^††,^***	600 mg every 12 hours orally
Clarithromycin^††,†††^	500 mg every 12 hours orally (only initiate after at least 3 days of treatment with any of the other antimicrobial drugs listed)
Dalbavancin^††^	1 g x 1 dose IV, then 500 mg weekly IV
**Antitoxin (only to be used if antimicrobial drugs are not available or not appropriate; listed antitoxins joined by “or” are considered equivalent)**	
Raxibacumab^§§§^	40 mg/kg in a single dose IV
or	
Obiltoxaximab^§§§^	16 mg/kg in a single dose IV

**Abbreviations:** FDA = Food and Drug Administration; IV = intravenous; PCN-S = penicillin-susceptible strains; PEP = postexposure prophylaxis; PEPAbx = antimicrobial postexposure prophylaxis for anthrax.

* Definitive therapy should be directed by antibiotic susceptibility test results, when available.

^†^ For pregnant adolescents, refer to pediatric guidelines for weight-based dosing (see Table 12).

^§^ Dosing recommended for pregnant persons regardless of trimester of infection.

^¶^ Approved by FDA for anthrax PEPAbx, treatment, or both, but specific uses (e.g., doses, dosing schedules, and patient populations) recommended in this report might differ from the FDA-approved labeling.

** If liquid formulations are not available for adults who cannot swallow pills, instructions are available for preparing oral suspensions of moxifloxacin (**Source:** Hutchinson DJ, Johnson CE, Klein KC. Stability of extemporaneously prepared moxifloxacin oral suspensions. Am J Health Syst Pharm 2009;66:665–7.121) and doxycycline (**Source:** CDC. In an anthrax emergency: how to prepare doxycycline hyclate for children and adults who cannot swallow pills. Atlanta, GA: US Department of Health and Human Services, CDC; 2020. https://www.cdc.gov/anthrax/public-health/doxy-crushing-instruction-pamphlet.html).

^††^ Not approved by FDA for anthrax PEPAbx or treatment.

^§§^ Ampicillin 500 mg every 6 hours can be used as an alternative to amoxicillin, if available.

^¶¶^ Alternative selections are for patients who have contraindications to or cannot tolerate first-line antimicrobial drugs or if first-line antimicrobial drugs are not available.

*** Linezolid can be considered for PEP in scenarios when patients can receive regular laboratory testing to monitor for myelosuppression or neurotoxicity, which might occur within 14–28 days of use. If possible, switch to a different drug when available.

^†††^ Clarithromycin is unlikely to be effective if the patient has bacteremia, thus a different antimicrobial must be used initially to clear bacteremia.

^§§§^ Premedicate with IV or oral diphenhydramine within 1 hour before administration. Hypersensitivity and anaphylaxis have been reported after raxibacumab and obiltoxaximab administration.

**TABLE 10:** Empiric* treatment regimens for pregnant or lactating persons^†,§^ aged *>*18 years with cutaneous anthrax without signs and symptoms of meningitis, by descending order of preference — CDC recommendations, 2023

Treatment (listed drugs joined by “or” are considered equivalent)	Dosage
**First-line antimicrobial drug**	
Doxycycline^¶,^**	100 mg every 12 hours orally
or	
Ciprofloxacin^¶^	500 mg every 12 hours orally
or	
Levofloxacin^¶^	750 mg every 24 hours orally
PCN-S only:	
Amoxicillin^††,§§^	1 g every 8 hours orally
or	
Penicillin VK^††^	500 mg every 6 hours orally
**Alternative antimicrobial drug^¶¶^**	
Amoxicillin/clavulanate^††^	16:1 formulation (1 g/62.5 mg) in 2 tablets every 12 hours orally
or	
Amoxicillin/clavulanate^††^	7:1 formulation (875/125 mg) every 12 hours orally
Moxifloxacin**^,††^	400 mg every 24 hours orally
Ofloxacin^††^	400 mg every 12 hours orally
Clindamycin^††^	600 mg every 8 hours orally
Omadacycline^††^	450 mg every 24 hours orally x 2 days, then 300 mg every 24 hours orally
Linezolid^††^	600 mg every 12 hours orally
Clarithromycin^††,^***	500 mg every 12 hours orally (only initiate after at least 3 days of treatment with any of the other antimicrobial drugs listed)
Dalbavancin^††^	1 g x 1 dose IV, then 500 mg weekly IV
Imipenem/cilastatin^††^	1 g every 6 hours IV
or	
Meropenem^††^	2 g every 8 hours IV
Vancomycin^††^	15 mg/kg every 12 hours IV over a period of 1–2 hours (target AUC_24_ of 400–600 *µ*g x h/mL [preferred]; if AUC_24_ is not available, maintain serum trough concentrations of 15–20 *µ*g/mL)
**Antitoxin (only to be used if antimicrobial drugs are not available or not appropriate; listed antitoxins joined by “or” are considered equivalent)**	
Raxibacumab^†††^	40 mg/kg in a single dose IV
or	
Obiltoxaximab^†††^	16 mg/kg in a single dose IV
AIGIV^§§§^	420 units IV

**Abbreviations:** AIGIV = anthrax immunoglobulin intravenous; AUC_24_ = area under the concentration-time curve from 0 to 24 hours; FDA = Food and Drug Administration; IV = intravenous; PCN-S = penicillin-susceptible strains; PEPAbx = antimicrobial postexposure prophylaxis for anthrax.

* Definitive therapy should be directed by antibiotic susceptibility test results, when available.

^†^ For pregnant adolescents, refer to pediatric guidelines for weight-based dosing (see Table 13).

^§^ Dosing recommended for pregnant persons regardless of trimester.

^¶^ Approved by FDA for anthrax PEPAbx, treatment, or both, but specific uses (e.g., doses, dosing schedules, and patient populations) recommended in this report might differ from the FDA-approved labeling.

** If liquid formulations are not available for adults who cannot swallow pills, instructions are available for preparing oral suspensions of moxifloxacin (**Source:** Hutchinson DJ, Johnson CE, Klein KC. Stability of extemporaneously prepared moxifloxacin oral suspensions. Am J Health Syst Pharm 2009;66:665–7.121) and doxycycline (**Source:** CDC. In an anthrax emergency: how to prepare doxycycline hyclate for children and adults who cannot swallow pills. Atlanta, GA: US Department of Health and Human Services, CDC; 2020. https://www.cdc.gov/anthrax/public-health/doxy-crushing-instruction-pamphlet.html).

^††^ Not approved by FDA for anthrax PEPAbx or treatment.

^§§^ Ampicillin 500 mg every 6 hours orally can be used as an alternative to amoxicillin, if available.

^¶¶^ Alternative selections are for patients who have contraindications to or cannot tolerate first-line antimicrobial drugs or if first-line antimicrobial drugs are not available.

*** Clarithromycin is unlikely to be effective if the patient has bacteremia, thus a different antimicrobial drug must be used initially to clear bacteremia.

^†††^ Premedicate with IV or oral diphenhydramine within 1 hour before administration. Hypersensitivity and anaphylaxis have been reported after raxibacumab and obiltoxaximab administration.

^§§§^ An 840-unit dose of AIGIV can be considered for severe cases.

**TABLE 11:** Empiric* treatment regimens for pregnant or lactating persons aged ≥18 years^†,§^ with systemic^¶^ anthrax with or without meningitis, by descending order of preference — CDC recommendations, 2023

Regimen		Example	
Regimen 1. Two bactericidal drugs from different antimicrobial drug classes plus a PSI or an RNAI		Ciprofloxacin plus meropenem plus doxycycline or omadacycline**	
Regimen 2. One bactericidal drug plus a PSI		Meropenem plus linezolid or doxycycline	
Regimen 3. One bactericidal drug plus a second bactericidal drug from a different antimicrobial drug class		Meropenem plus ciprofloxacin	
Regimen 4. One bactericidal drug plus an RNAI (rifampin should not be used as monotherapy)		Meropenem plus rifampin	
Regimen 5. A PSI plus an RNAI (rifampin should not be used as monotherapy)		Linezolid or doxycycline or clindamycin plus rifampin	
Regimen 6. Two PSIs from different antimicrobial drug classes		Linezolid plus doxycycline	
Regimen 7. A single bactericidal drug		Meropenem	
Regimen 8. A single PSI		Linezolid or doxycycline or clindamycin	
**First-line antimicrobial drug^††^**			
**Bactericidal drug**		**PSI**	
**Treatment (listed drugs joined by “or” are considered equivalent)**	**Dosage**	**Treatment**	**Dosage**
Meropenem^§§^	2 g every 8 hours IV	Doxycycline^¶¶^	200 mg x 1 dose IV, then 100 mg every 12 hours IV
or			
Ciprofloxacin^¶¶^	400 mg every 8 hours IV		
or			
Levofloxacin^¶¶^	500 mg every 12 hours IV		
PCN-S only:			
Penicillin G^¶¶^	4 million units every 4 hours IV		
or			
Ampicillin^§§^	2 g every 4 hours IV		
Imipenem/cilastatin^§§^	1 g every 6 hours IV		
or			
Ampicillin/sulbactam^§§^	3 g every 6 hours IV		
Alternative antimicrobial drug***			
**Bactericidal drug**		**PSI/RNAI**	
**Treatment**	**Dosage**	**Treatment**	**Dosage**
Piperacillin/tazobactam^§§^	3.375 g every 4 hours IV	Omadacycline^§§,†††^	200 mg x 1 dose IV on day 1, then 100 mg every 24 hours IV
Moxifloxacin^§§^	400 mg every 24 hours IV	Eravacycline^§§,†††^	1 mg/kg every 12 hours IV
Vancomycin^§§,†††^	15 mg/kg every 12 hours IV over a period of 1–2 hours (target AUC_24_ of 400–600 *µ*g x h/mL [preferred]; if AUC_24_ is not available, maintain serum trough concentrations of 15–20 *µ*g/mL). Consider a loading dose of 20-35 mg/kg for critically ill patients.	Clindamycin^§§^	900 mg every 8 hours IV
		Linezolid^§§^	600 mg every 12 hours IV
		Rifampin^§§,§§§^	600 mg every 12 hours IV
plus			
**Antitoxin (single dose as an adjunct to antimicrobial drug; listed antitoxins joined by “or” are considered equivalent)**			
**Treatment**		**Dosage**	
Raxibacumab^¶¶¶^		40 mg/kg IV	
or			
Obiltoxaximab^¶¶¶^		16 mg/kg IV	
AIGIV		420 units IV	

**Abbreviations:** AIGIV = anthrax immunoglobulin intravenous; AUC_24_ = area under the concentration-time curve from 0 to 24 hours; FDA = Food and Drug Administration; IV = intravenous; PCN-S = penicillin-susceptible strains; PEPAbx = antimicrobial postexposure prophylaxis for anthrax; PSI = protein synthesis inhibitor; RNAI = RNA synthesis inhibitor.

* Definitive therapy should be directed by antibiotic susceptibility test results, when available.

^†^ Pregnant adolescents: refer to pediatric guidelines for weight-based dosing (see Table 14).

^§^ Dosing recommended for pregnant persons regardless of trimester. If meningitis is not suspected and susceptibilities are known, start at regimen 2.

^¶^ “Systemic” was defined as including evidence of organ damage or any of the following: hyperthermia or hypothermia, tachycardia, tachypnea, hypotension, or leukocytosis or leukopenia (**Source:** Katharios-Lanwermeyer S, Holty JE, Person M, et al. Identifying meningitis during an anthrax mass casualty incident: systematic review of systemic anthrax since 1880. Clin Infect Dis 2016;62:1537–45).

** For anthrax meningitis, consider using antimicrobial drugs that have demonstrated potential neuroprotective benefits in vivo (e.g., minocycline, doxycycline, clindamycin, and ß-lactamase inhibitors).

^††^ For highly bioavailable antimicrobial drugs (e.g., ciprofloxacin, doxycycline, and linezolid), if the IV formulation is not available, oral formulations can be considered for patients with an intact gastrointestinal tract where absorption is expected to be complete after oral administration.

^§§^ Not approved by FDA for anthrax PEPAbx or treatment.

^¶¶^ Approved by FDA for anthrax PEPAbx, treatment, or both, but specific uses (e.g., doses, dosing schedules, and patient populations) recommended in this report might differ from the FDA-approved labeling.

*** Alternative selections are for patients who have contraindications to or cannot tolerate first-line antimicrobial drugs or if first-line antimicrobial drugs are not available.

^†††^ This antimicrobial does not cross an intact blood-brain barrier but can cross with meningitis because of breakdown of the barrier.

^§§§^ Rifampin is an RNAI and also bactericidal; however, it should not be used as monotherapy.

^¶¶¶^ Premedicate with IV or oral diphenhydramine within 1 hour before administration. Hypersensitivity and anaphylaxis have been reported after raxibacumab and obiltoxaximab administration.

**** An 840-unit dose of AIGIV can be considered for severe cases.

##### PEP and Treatment Regimens for Cutaneous Anthrax Without Signs and Symptoms of Meningitis

For pregnant and lactating persons aged ≥18 years, empiric PEP (Table 9) and empiric cutaneous anthrax treatment (Table 10) regimens include either a single antimicrobial drug or a single antitoxin. These regimens are summarized as follows:

- Antimicrobial drug: Choose a single antimicrobial drug.
  º Antimicrobial drugs are listed in descending order of preference in the table. Listed drugs joined by “or” are considered equivalent.
  ° Continue or switch antimicrobial drug based on susceptibility testing once available.
  ° Only choose a “PCN-S only” antimicrobial drug after the strain has been determined to be penicillin susceptible.
- Antitoxin: Choose a single antitoxin if no antimicrobial drugs are available.

For all pregnant and lactating persons who have an aerosol exposure (e.g., a bioterrorism-related incident or animal skin drum–related event), PEPAbx should continue for 60 days from the exposure whether or not vaccine is given (*33*). For nonaerosol (i.e., cutaneous or ingestion) exposures, PEPAbx should continue for 7 days and vaccine is not needed. 

For pregnant and lactating persons with cutaneous anthrax without signs and symptoms of meningitis, the treatment regimen should continue for 7–10 days, or until clinical criteria for stability are met. If an aerosol exposure might have occurred, patients should transition from a treatment to a PEP regimen (Table 9); the combined regimen should total 60 days from exposure. If no aerosolizing event occurred, patients with cutaneous anthrax do not need to continue PEPAbx.

##### Treatment Regimens for Systemic Anthrax With or Without Meningitis

For pregnant or lactating persons aged ≥18 years, empiric treatment regimens for those with systemic anthrax with or without meningitis (Table 11) summarized as follows:

- Antimicrobial drugs: Choose two bactericidal drugs from different antimicrobial drug classes plus a PSI or an RNAI.
  º Antimicrobial drugs are listed in descending order of preference in the table. Listed drugs joined by “or” are considered equivalent.
  º Continue or switch antimicrobial drugs based on susceptibility testing once available.
  º Only choose a “PCN-S only” antimicrobial drug after the strain has been determined to be penicillin susceptible.
- Antitoxin: Choose a single antitoxin as adjunctive therapy.

If an appropriate combination of bactericidal drugs plus a PSI or an RNAI is contraindicated, not well tolerated, or not available or if meningitis is considered unlikely, consider the following regimens in descending order of preference:

- One bactericidal drug plus a PSI (start with this regimen if meningitis is not suspected and susceptibilities are known)
- One bactericidal drug plus a second bactericidal drug from a different antimicrobial drug class
- One bactericidal drug plus an RNAI
- A PSI plus an RNAI
- Two PSIs from different antimicrobial drug classes
- A single bactericidal drug
- A single PSI

From a PK/PD perspective, minocycline and doxycycline are the preferred PSIs because they provide more robust drug exposures in plasma and CSF compared with macrolides or clindamycin. A single RNAI (i.e., rifampin) should not be used as monotherapy because of the potential for rapid development of resistance (*135*). In addition, when meningitis is not suspected, certain oral formulations are included as alternatives in case IV formulations are not available.

Duration of antimicrobial drug treatment should be for 2 weeks or longer; however, duration can be shortened and IV administration transitioned to oral medication based on patient improvement and clinical judgment. Patients with naturally acquired noninhalation cases do not need continuation of antimicrobial drugs for PEP. If an aerosol exposure might have occurred (i.e., a bioterrorism-related incident or animal skin drum–related event), healthy patients treated for systemic disease need no further antimicrobial drugs for PEP because they will have developed natural immunity. However, patients with compromised immune systems should transition to an oral PEP regimen (Table 9). The total duration of antimicrobial drugs (i.e., treatment plus PEP) should be 60 days from onset of illness.

#### Children Aged ≥1 Month to <18 Years

The PEP and treatment guidelines for children aged ≥1 month to <18 years were based on those for nonpregnant adults aged ≥18 years. Thus, the recommendations for children are similar to those for nonpregnant adults. This principle also applies for empiric PEP (Table 12), empiric treatment of cutaneous anthrax without signs and symptoms of meningitis (Table 13), and empiric treatment of systemic anthrax with or without meningitis (Table 14). The pediatric recommendations differ from the adult recommendations because of the potential for adverse events related to the recommended antimicrobial drugs.

**TABLE 12:** Empiric* postexposure prophylaxis for children aged ≥1 month to <18 years after exposure to *Bacillus anthracis*, by descending order of preference — CDC recommendations, 2023

Treatment (listed drugs joined by “or” are considered equivalent)	Dosage
**First-line antimicrobial drug**	
Ciprofloxacin^†^	15 mg/kg every 12 hours orally (maximum 500 mg/dose)
or	
Doxycycline^†,§^	<45 kg: 2.2 mg/kg every 12 hours orally (maximum 100 mg/dose) ≥45 kg: 100 mg every 12 hours orally
or	
Levofloxacin^†^	<50 kg: 8 mg/kg every 12 hours orally (maximum 250 mg/dose) ≥50 kg: 500 mg every 24 hours orally
PCN-S only^¶^:	
Amoxicillin^**,††^	25 mg/kg every 8 hours orally (maximum 500 mg/dose)
Penicillin VK^**^	12.5–18.7 mg/kg every 6 hours orally (maximum 500 mg/dose)
Amoxicillin/clavulanate**^,§§^	Aged ≥3 months: 7:1 formulation (200/28.5 mg or 400/57 mg) 22.5 mg/kg based on amoxicillin component every 12 hours orally (maximum 875/125 mg/dose) Aged ≥3 months and <40 kg: 14:1 formulation (600/42.9 mg) 45 mg/kg based on amoxicillin component every 12 hours orally Aged ≥3 months and ≥40 kg: 16:1 formulation (1,000/62.5 mg tablets) 2 g based on amoxicillin component every 12 hours orally
Clindamycin**	10 mg/kg every 8 hours orally (maximum 600 mg/dose)
**Alternative antimicrobial drug^¶¶^**	
Moxifloxacin^§,^***^,†††^	Aged ≥3 to ≤23 months: 6 mg/kg every 12 hours orally (maximum 200 mg/dose) Aged 2 to <6 years: 5 mg/kg every 12 hours orally (maximum 200 mg/dose) Aged 6 to <12 years: 4 mg/kg every 12 hours orally (maximum 200 mg/dose) Aged ≥12 to <18 years and <45 kg: 4 mg/kg every 12 hours orally (maximum 200 mg/dose) Aged ≥12 to <18 years and ≥45 kg: 400 mg every 12 hours orally
Minocycline^†,§§§^	4 mg/kg once (maximum 200-mg dose) orally, then 2 mg/kg every 12 hours orally (maximum 100 mg/dose)
Ofloxacin***	11.25 mg/kg every 12 hours orally (maximum 400 mg/dose)
Tetracycline^†,§§§^	12.5 mg/kg every 6 hours orally (maximum 500 mg/dose)
Linezolid**^,¶¶¶^	Aged <12 years: 10 mg/kg every 8 hours orally (maximum 600 mg/dose) Aged ≥12 years: 600 mg every 12 hours orally
Omadacycline***^,§§§^	Aged >8 years: 450 mg every 24 hours orally x 2 days, then 300 mg every 24 hours orally
Clarithromycin**^,^****	7.5 mg/kg every 12 hours orally (maximum 500 mg/dose; only initiate after at least 3 days of treatment with any of the other antimicrobial drugs listed)
Dalbavancin**	Aged ≥3 months to <6 years: 22.5 mg/kg every 2 weeks IV (maximum 1.5 g/dose) Aged ≥6 years to <18 years: 18 mg/kg every 2 weeks IV (maximum 1.5 g/dose)
**Antitoxin (only to be used if antimicrobial drugs are not available or not appropriate; (listed antitoxins joined by “or” are considered equivalent)**	
Raxibacumab^††††^	≤10 kg: 80 mg/kg as a single dose IV >10 kg to 40 kg: 60 mg/kg as a single dose IV >40 kg: 40 mg/kg as a single dose IV
or	
Obiltoxaximab^††††^	≤15 kg: 32 mg/kg as a single dose IV >15 kg to 40 kg: 24 mg/kg as a single dose IV >40 kg: 16 mg/kg as a single dose IV

**Abbreviations:** FDA = Food and Drug Administration; PCN-S = penicillin-susceptible strains; IV = intravenous; PEP = postexposure prophylaxis; PEPAbx = antimicrobial postexposure prophylaxis for anthrax.

* Definitive therapy should be directed by antibiotic susceptibility test results, when available.

^†^ Approved by FDA for anthrax PEPAbx, treatment, or both, but specific uses (e.g., doses, dosing schedules, and patient populations) recommended in this report might differ from the FDA-approved labeling.

^§^ If liquid formulations are not available for children who cannot swallow pills, instructions are available for preparing oral suspensions of moxifloxacin (**Source:** Hutchinson DJ, Johnson CE, Klein KC. Stability of extemporaneously prepared moxifloxacin oral suspensions. Am J Health Syst Pharm 2009;66:665–7.121) and doxycycline (**Source:** CDC. In an anthrax emergency: how to prepare doxycycline hyclate for children and adults who cannot swallow pills. Atlanta, GA: US Department of Health and Human Services, CDC; 2020. https://www.cdc.gov/anthrax/public-health/doxy-crushing-instruction-pamphlet.html).

^¶^ Up to 10% of naturally occurring strains of anthrax are resistant to penicillin and amoxicillin; bioterror strains can be engineered to generate resistance to multiple antibiotics. Susceptibility results reported from CDC within 48–72 hours of initial isolation of anthrax.

** Not approved by FDA for anthrax PEPAbx or treatment.

^††^ Ampicillin 25 mg/kg every 6 hours orally (maximum 500 mg/dose) can be used as an alternative to amoxicillin, if available.

^§§^ To minimize potential side effects from clavulanate, the amoxicillin/clavulanate 875/125 mg every 12 hours orally and amoxicillin/clavulanate extended-release 2,000/125 mg every 12 hours orally treatments are preferred over 500/125 mg every 8 hours orally.

^¶¶^ Alternative selections are for patients who have contraindications to or cannot tolerate first-line antimicrobial drugs or if first-line antimicrobial drugs are not available.

*** Not approved by FDA for any pediatric indications. Not approved by FDA for anthrax PEPAbx or treatment.

^†††^ For children aged 12–17 years who weigh ≥45 kg with risk factors for cardiac events, consider 200 mg twice daily.

^§§§^ The use of tetracycline-class antibiotics during tooth development (i.e., last half of pregnancy, infancy, and childhood up to age 8 years) might cause permanent discoloration of the teeth (yellow, gray, or brown) and enamel hypoplasia. This adverse effect appears to occur less often with doxycycline but might occur more with longer durations of therapy.

^¶¶¶^ Linezolid can be considered for PEP in scenarios when patients can receive regular laboratory testing to monitor for myelosuppression or neurotoxicity, which might occur within 14–28 days of use. If possible, switch to a different drug when available.

**** Clarithromycin is unlikely to be effective if the patient has bacteremia, thus a different antimicrobial drug must be used initially to clear bacteremia.

^††††^ Premedicate with IV or oral diphenhydramine within 1 hour before administration. Hypersensitivity and anaphylaxis have been reported after raxibacumab and obiltoxaximab administration.

**TABLE 13:** Empiric* treatment regimens for children aged ≥1 month to <18 years with cutaneous anthrax without signs and symptoms of meningitis, by descending order of preference — CDC recommendations, 2023

Treatment (listed drugs joined by “or” are considered equivalent)	Dosage
**First-line antimicrobial drug**	
Ciprofloxacin^†^	15 mg/kg every 12 hours orally (maximum 500 mg/dose)
or	
Levofloxacin^†^	<50 kg: 8 mg/kg every 12 hours orally (maximum 250 mg/dose) ≥50 kg: 750 mg every 24 hours orally
or	
Doxycycline^†,§^	<45 kg: 2.2 mg/kg every 12 hours orally (maximum 100 mg/dose) ≥45 kg: 100 mg every 12 hours orally
or	
Minocycline^†,¶^	4 mg/kg once orally (maximum 200 mg/dose), then 2 mg/kg every 12 hours orally (maximum 100 mg/dose)
PCN-S only:	
Amoxicillin**^,††^	25 mg/kg every 8 hours orally (maximum 1 g/dose)
or	
Penicillin VK**	12.5–18.7 mg/kg every 6 hours orally (maximum 500 mg/dose)
Amoxicillin/clavulanate**^,§§^	Aged ≥3 months: 7:1 formulation (200/28.5 mg or 400/57 mg) 22.5 mg/kg based on amoxicillin component every 12 hours orally (maximum 875/125 mg/dose) Aged ≥3 months and <40 kg: 14:1 formulation (600/42.9 mg) 45 mg/kg based on amoxicillin component every 12 hours orally Aged ≥3 months and ≥40 kg: 16:1 formulation (1,000/62.5 mg tablets) 2 g every 12 hours orally
Clindamycin**	10 mg/kg every 8 hours orally (maximum 600 mg/dose)
**Alternative antimicrobial drug^¶¶^**	
Moxifloxacin^§,^***^,†††^	Aged ≥3 to ≤23 months: 6 mg/kg every 12 hours orally (maximum 200 mg/dose) Aged 2 to <6 years: 5 mg/kg every 12 hours orally (maximum 200 mg/dose) Aged 6 to <12 years: 4 mg/kg every 12 hours orally (maximum 200 mg/dose) Aged ≥12 to <18 years and <45 kg: 4 mg/kg every 12 hours orally (maximum 200 mg/dose) Aged ≥12 to <18 years and ≥45 kg: 400 mg every 24 hours orally
Ofloxacin***	11.25 mg/kg every 12 hours orally (maximum 400 mg/dose)
Tetracycline^†,¶^	12 mg/kg every 6 hours orally (maximum 500 mg/dose)
Linezolid**	Aged <12 years: 10 mg/kg every 8 hours orally (maximum 600 mg/dose) Aged ≥12 years: 600 mg every 12 hours orally
Omadacycline^¶,^***	Aged >8 years: 450 mg every 24 hours orally x 2 days, then 300 mg every 24 hours orally
Clarithromycin**^,§§§^	7.5 mg/kg every 12 hours orally (maximum 500 mg/dose; only initiate after at least 3 days of treatment with any of the other antimicrobial drugs listed)
Dalbavancin**	Aged ≥3 months to <6 years: 22.5 mg/kg every week IV (maximum 1.5 g/dose) Aged ≥6 years to <18 years: 18 mg/kg every week IV (maximum 1.5 g/dose)
Meropenem**	20 mg/kg every 8 hours IV (maximum 2 g/dose)
Imipenem/cilastatin**	25 mg/kg every 6 hours IV (maximum 1 g/dose)
Vancomycin**	20 mg/kg every 8 hours IV over a period of 1–2 hours (target AUC_24_ of 400 *µ*g x h/mL [preferred]; if AUC_24_ is not available, maintain serum trough concentrations of 15–20 *µ*g/mL)
**Antitoxin (only to be used if antimicrobial drugs are not available or not appropriate; listed antitoxins joined by “or” are considered equivalent)**	
Raxibacumab^¶¶¶^	≤10 kg: 80 mg/kg as a single dose IV >10–40 kg: 60 mg/kg as a single dose IV >40 kg: 40 mg/kg as a single dose IV
or	
Obiltoxaximab^¶¶¶^	≤15 kg: 32 mg/kg as a single dose IV >15–40 kg: 24 mg/kg as a single dose IV >40 kg: 16 mg/kg as a single dose IV
AIGIV****	<10 kg: 1 vial (approximately 60 units) IV 10 to <18 kg: 2 vials (approximately 120 units) IV 18 to <25 kg: 3 vials (approximately 180 units) IV 25 to <35 kg: 4 vials (approximately 240 units) IV 35 to <50 kg: 5 vials (approximately 300 units) IV 50 to <60 kg: 6 vials (approximately 360 units) IV ≥60 kg: 7 vials (approximately 420 units) IV

**Abbreviations:** AUC_24_ = area under the concentration-time curve from 0 to 24 hours; FDA = Food and Drug Administration; IV = intravenous; PCN-S = penicillin-susceptible strains; PEPAbx = antimicrobial postexposure prophylaxis for anthrax.

* Definitive therapy should be directed by antibiotic susceptibility test results, when available.

^†^ Approved by FDA for anthrax PEPAbx, treatment, or both, but specific uses (e.g., doses, dosing schedules, and patient populations) recommended in this report might differ from the FDA-approved labeling.

^§^ Instructions are available for preparing oral suspensions of moxifloxacin (**Source:** Hutchinson DJ, Johnson CE, Klein KC. Stability of extemporaneously prepared moxifloxacin oral suspensions. Am J Health Syst Pharm 2009;66:665–7.121) and doxycycline (**Source:** CDC. In an anthrax emergency: how to prepare doxycycline hyclate for children and adults who cannot swallow pills. Atlanta, GA: US Department of Health and Human Services, CDC; 2020. https://www.cdc.gov/anthrax/public-health/doxy-crushing-instruction-pamphlet.html).

^¶^ The use of tetracycline-class antibiotics during tooth development (i.e., last half of pregnancy, infancy, and childhood up to age 8 years) might cause permanent discoloration of the teeth (yellow, gray, or brown) and enamel hypoplasia. This adverse effect appears to occur less often with doxycycline but might occur more with longer durations of therapy.

** Not approved by FDA for anthrax PEPAbx or treatment.

^††^ Ampicillin 25 mg/kg every 6 hours orally (maximum 500 mg/dose) can be used as an alternative to amoxicillin, if available.

^§§^ To minimize potential side effects from clavulanate, the amoxicillin/clavulanate 875/125 mg every 12 hours orally and amoxicillin/clavulanate extended-release 2,000/125 mg every 12 hours orally treatments are preferred over 500/125 mg every 8 hours orally.

^¶¶^ Alternative selections are for patients who have contraindications to or cannot tolerate first-line antimicrobial drugs or if first-line antimicrobial drugs are not available.

*** Not approved by FDA for any pediatric indications. Not approved by FDA for anthrax PEPAbx or treatment.

^†††^ For children aged 12–17 years who weigh ≥45 kg with risk factors for cardiac events, consider 200 mg twice daily.

^§§§^ Clarithromycin is unlikely to be effective if the patient has bacteremia, thus a different antimicrobial drug must be used initially to clear bacteremia.

^¶¶¶^ Premedicate with IV or oral diphenhydramine within 1 hour before administration. Hypersensitivity and anaphylaxis have been reported after raxibacumab and obiltoxaximab administration.

**** Dose can be doubled for severe cases in patients who weigh >5 kg.

**TABLE 14:** Empiric* treatment regimens for children aged ≥1 month to <18 years with systemic^†^ anthrax with or without meningitis,^§^ by descending order of preference — CDC recommendations, 2023

Regimen		Example	
Regimen 1. Two bactericidal drugs from different antimicrobial drug classes plus a PSI or an RNAI		Ciprofloxacin plus meropenem plus linezolid or minocycline^¶^	
Regimen 2. One bactericidal drug plus a PSI		Meropenem plus linezolid or doxycycline	
Regimen 3. One bactericidal drug plus a second bactericidal drug from a different antimicrobial drug class		Meropenem plus ciprofloxacin	
Regimen 4. One bactericidal drug plus an RNAI (rifampin should not be used as monotherapy)		Meropenem plus rifampin	
Regimen 5. A PSI plus an RNAI (rifampin should not be used as monotherapy)		Doxycycline or chloramphenicol or linezolid plus rifampin	
Regimen 6. Two PSIs from different antimicrobial drug classes		Doxycycline plus chloramphenicol	
Regimen 7. A single bactericidal drug		Meropenem	
Regimen 8. A single PSI		Doxycycline or chloramphenicol or linezolid	
First-line antimicrobial drug**			
**Bactericidal drug**		**PSI**	
**Treatment (listed drugs joined by “or” are considered equivalent)**	**Dosage**	**Treatment**	**Dosage**
Meropenem^††^	40 mg/kg every 8 hours IV (maximum 2 g/dose)	Minocycline^§§,¶¶^	4 mg/kg once IV (maximum 200 mg dose), then 2 mg/kg every 12 hours IV (maximum 100 mg/dose)
or		Doxycycline^§§^	<45 kg: 2.2 mg/kg loading dose IV (maximum 200 mg/dose), then 2.2 mg/kg every 12 hours IV (maximum 100 mg/dose) ≥45 kg: 200 mg IV loading dose, then 100 mg every 12 hours IV
Ciprofloxacin^§§^	10 mg/kg every 8 hours IV (maximum 400 mg/dose)		
or			
Levofloxacin^§§^	<50 kg: 10 mg/kg every 12 hours IV (maximum 250 mg/dose) ≥50 kg: 750 mg every 24 hours IV		
PCN-S only:			
Ampicillin^††^	50 mg/kg every 6 hours IV (maximum 3 g/dose)		
or			
Penicillin G^§§^	67,000 units/kg every 4 hours IV (maximum 4 million units/dose)		
Alternative antimicrobial drug***			
**Bactericidal drug**		**PSI/RNAI**	
**Treatment (listed drugs joined by “or” are considered equivalent)**	**Dosage**	**Treatment**	**Dosage**
Imipenem/cilastatin^††,†††^	25 mg/kg every 6 hours IV (maximum 1 g/dose)	Clindamycin^††^	13.3 mg/kg every 8 hours IV (maximum 900 mg/dose)
Piperacillin/tazobactam^††^	75 mg piperacillin/kg every 6 hours IV (maximum 4 g piperacillin/dose)	Eravacycline^¶¶,§§§^	Aged >8 years: 1 mg/kg every 12 hours IV
or		Linezolid^††^	Aged <12 years: 10 mg/kg every 8 hours IV (maximum 600 mg/dose) Aged ≥12 years: 15 mg/kg every 12 hours IV (maximum 600 mg/dose)
Ampicillin/sulbactam^††^	50 mg ampicillin/kg every 6 hours IV (maximum 2 g ampicillin/dose)		
Moxifloxacin^§§§,¶¶¶^	Aged ≥3 to ≤23 months: 6 mg/kg every 12 hours IV (maximum 200 mg/dose) Aged 2 to <6 years: 5 mg/kg every 12 hours IV (maximum 200 mg/dose) Aged 6 to <12 years: 4 mg/kg every 12 hours (maximum 200 mg/dose) Aged ≥12 to ≤18 years and <45 kg: 4 mg/kg every 12 hours IV (maximum 200 mg/dose) Aged ≥12 to ≤18 years and ≥45 kg: 400 mg every 24 hours IV	Rifampin**^,^****	10 mg/kg every 12 hours IV (maximum 300 mg/dose)
Vancomycin^††^	20 mg/kg every 8 hours IV (target AUC_24_ of 400 *µ*g x h/mL [preferred]; if AUC_24_ is not available, maintain serum trough concentrations of 15–20 *µ*g/mL)	Chloramphenicol^††,††††^	25 mg/kg every 6 hours IV (maximum 1 g/dose)
plus			
**Antitoxin (single dose as an adjunct to antimicrobial drug)**			
**Treatment (listed antitoxins joined by “or” are considered equivalent)**		**Dosage**	
Raxibacumab^§§§§^		≤10 kg: 80 mg/kg as a single dose IV >10 to 40 kg: 60 mg/kg as a single dose IV >40 kg: 40 mg/kg as a single dose IV	
or			
Obiltoxaximab^§§§§^		≤15 kg: 32 mg/kg as a single dose IV >15 to 40 kg: 24 mg/kg as a single dose IV >40 kg: 16 mg/kg as a single dose IV	
AIGIV^¶¶¶¶^		<10 kg: 1 vial (approximately 60 units) IV 10 to <18 kg: 2 vials (approximately 120 units) IV 18 to <25 kg: 3 vials (approximately 180 units) IV 25 to <35 kg: 4 vials (approximately 240 units) IV 35 to <50 kg: 5 vials (approximately 300 units) IV 50 to <60 kg: 6 vials (approximately 360 units) IV ≥60 kg: 7 vials (approximately 420 units) IV	

**Abbreviations:** AIGIV = anthrax immunoglobulin intravenous; AUC_24_ = area under the concentration-time curve from 0 to 24 hours; FDA = Food and Drug Administration; IV = intravenous; PCN-S = penicillin-susceptible strains; PEPAbx = antimicrobial postexposure prophylaxis for anthrax; PSI = protein synthesis inhibitor; RNAI = RNA synthesis inhibitor.

* Definitive therapy should be directed by antibiotic susceptibility test results, when available.

^†^ “Systemic” was defined as including evidence of organ damage or any of the following: hyperthermia or hypothermia, tachycardia, tachypnea, hypotension, or leukocytosis or leukopenia (**Source:** Katharios-Lanwermeyer S, Holty JE, Person M, et al. Identifying meningitis during an anthrax mass casualty incident: systematic review of systemic anthrax since 1880. Clin Infect Dis 2016;62:1537–45).

^§^ If meningitis is not suspected and susceptibilities are known, start at regimen 2.

^¶^ For anthrax meningitis, consider using antimicrobial drugs that have demonstrated potential neuroprotective benefits in vivo (e.g., minocycline, doxycycline, clindamycin, and ß-lactam antimicrobials).

** For highly bioavailable antimicrobial drugs (e.g., ciprofloxacin, doxycycline, and linezolid), if the IV formulation is not available, oral formulations can be considered for patients with an intact gastrointestinal tract where absorption is expected to be complete after oral administration.

^††^ Not approved by FDA for anthrax PEPAbx or treatment.

^§§^ Approved by FDA for anthrax PEPAbx, treatment, or both, but specific uses (e.g., doses, dosing schedules, and patient populations) recommended in this report might differ from the FDA-approved labeling.

^¶¶^ The use of tetracycline-class antibiotics during tooth development (i.e., last half of pregnancy, infancy, and childhood up to age 8 years) might cause permanent discoloration of the teeth (yellow, gray, or brown) and enamel hypoplasia. This adverse effect appears to occur less often with doxycycline but might occur more with longer durations of therapy.

*** Alternative selections are for patients who have contraindications to or cannot tolerate first-line antimicrobial drugs or if first-line antimicrobial drugs are not available.

^†††^ Increased seizure risk if meningitis/central nervous system anthrax is present.

^§§§^ Not approved by FDA for any pediatric indications. Not approved by FDA for anthrax PEPAbx or treatment.

^¶¶¶^ For children aged 12–17 years who weigh ≥45 kg with risk factors for cardiac events, consider 200 mg twice daily.

**** Rifampin is an RNAI and also bactericidal; however, it should not be used as monotherapy.

^††††^ Chloramphenicol should not be used in combination with a bactericidal antimicrobial drug because the interaction might be antagonistic.

^§§§§^ Premedicate with IV or oral diphenhydramine within 1 hour before administration. Hypersensitivity and anaphylaxis have been reported after raxibacumab and obiltoxaximab administration.

^¶¶¶¶^ Dose can be doubled for severe cases in patients who weigh >5 kg.

##### PEP and Treatment Regimens for Cutaneous Anthrax Without Signs and Symptoms of Meningitis 

For children aged ≥1 month to <18 years, empiric PEP (Table 12) and empiric cutaneous anthrax treatment (Table 13) regimens include either a single antimicrobial drug or a single antitoxin. These regimens are summarized as follows:

- Antimicrobial drug: Choose a single antimicrobial drug.
  ° Antimicrobial drugs are listed in descending order of preference in the table. Listed drugs joined by “or” are considered equivalent.
  ° Continue or switch antimicrobial drug based on susceptibility testing once available.
  ° Only choose a “PCN-S only” antimicrobial drug after the strain has been determined to be penicillin susceptible.
  º If the strain is found to be penicillin susceptible, a penicillin-class antimicrobial drug is preferred for first-line therapy.
  º For penicillin-resistant strains of anthrax, the benefits of therapy with fluoroquinolones and tetracyclines for pediatric anthrax far exceed the potential toxicities.
- Antitoxin: Choose a single antitoxin if no antimicrobial drugs are available.

For all children aged <18 years who have an aerosol exposure (e.g., a bioterrorism-related incident or animal skin drum–related event), PEPAbx should continue for 60 days from the exposure whether or not vaccine is given (*33*). For nonaerosol (i.e., cutaneous or ingestion) exposures, PEPAbx should continue for 7 days and vaccine is not needed. 

 For all children aged <18 years with cutaneous anthrax without signs and symptoms of meningitis, the treatment regimen should continue for 7–10 days, or until clinical criteria for stability are met. If an aerosol exposure might have occurred, patients should transition from a treatment to a PEP regimen (Table 12); the combined regimen should total 60 days from exposure. If no aerosolizing event occurred, patients with cutaneous anthrax do not need to continue PEPAbx.

##### Treatment Regimens for Systemic Anthrax With or Without Meningitis

 Empiric treatment regimens for children aged >1 month to <18 years with systemic anthrax with or without meningitis (Table 14) are summarized as follows:

- Antimicrobial drugs: Choose two bactericidal drugs from different antimicrobial drug classes plus a PSI or an RNAI.
  º Antimicrobial drugs are listed in descending order of preference in the table. Listed drugs joined by “or” are considered equivalent.
  º Continue or switch antimicrobial drugs based on susceptibility testing once available.
  º Only choose a “PCN-S only” antimicrobial drug after the strain has been determined to be penicillin susceptible.
- Antitoxin: Choose a single antitoxin as adjunctive therapy.

If an appropriate combination of bactericidal drug plus a PSI or an RNAI is contraindicated, not well tolerated, or not available for treatment of noncutaneous systemic anthrax, consider the following regimens in descending order of preference:

- One bactericidal drug plus a PSI (start with this regimen if meningitis is not suspected)
- One bactericidal drug plus a second bactericidal agent from a different antimicrobial drug class
- One bactericidal drug plus an RNAI
- A PSI plus an RNAI
- Two PSIs from different antimicrobial drug classes
- A single bactericidal drug
- A single PSI

From a PK/PD perspective, minocycline and doxycycline are the preferred PSIs because they provide more robust drug exposures in plasma and CSF compared with macrolides or clindamycin. A single RNAI (i.e., rifampin) should not be used as monotherapy because of the potential for rapid development of resistance (*135*). When meningitis is not suspected, certain oral formulations are included as alternatives in case IV formulations are not available.

Duration of antimicrobial drug treatment should be for 2 weeks or longer; however, duration can be shortened and IV administration transitioned to oral medication based on patient improvement and clinical judgment. Patients with naturally acquired noninhalation anthrax do not need continuation of antimicrobial drug therapy for PEP. If an aerosol exposure might have occurred (i.e., a bioterrorism-related incident or animal skin drum–related event), patients who are immunocompetent do not need further antimicrobial drug therapy because they will have developed natural immunity. Patients who are immunocompromised should transition to an oral PEP regimen (Table 12). The total duration of antimicrobial drug therapy (i.e., treatment plus PEP) should be 60 days from onset of illness.

#### Preterm and Full-Term Newborns

Virtually no data are available on antimicrobial drug dosing in neonates and premature infants. Dosing guidance for anthrax in newborn infants is based on extrapolation of data from older populations by using pharmacologic data modeling that incorporates antimicrobial drug kinetics, safety, and efficacy in newborns and how the broad range of developmental changes in this immature population affects therapy (*32**,**141**–**171*). Recommendations for both preterm and full-term newborns 32–44 weeks’ postmenstrual age (i.e., gestational age plus chronologic age) are available for empiric PEP (Table 15), empiric treatment of cutaneous anthrax without signs and symptoms of meningitis (Table 16), and empiric treatment of systemic anthrax with or without meningitis (Table 17). For neonates of earlier gestational age or without developmentally appropriate renal and hepatic function, providers should consult with a neonatologist, pharmacologist, or infectious diseases physician for appropriate dosing.

**TABLE 15:** Empiric* postexposure prophylaxis for preterm and full-term neonates 32–44 weeks’ postmenstrual age (gestational age plus chronologic age) after exposure to *Bacillus anthracis*, by descending order preference — CDC recommendations, 2023

Treatment	32 to <34 weeks’ gestational age		34 to <37 weeks’ gestational age		Full-term infant	
	0 to <1 week	1–4 weeks	0 to <1 week	1–4 weeks	0 to <1 week	1–4 weeks
	Dosage	Dosage	Dosage	Dosage	Dosage	Dosage
**First-line antimicrobial drug**						
PCN-S only^†^:						
Amoxicillin^§,¶^	25 mg/kg every 12 hours orally	25 mg/kg every 8 hours orally	25 mg/kg every 8 hours orally	25 mg/kg every 8 hours orally	25 mg/kg every 8 hours orally	25 mg/kg every 8 hours orally
Penicillin VK^§^	25 mg/kg every 12 hours orally	25 mg/kg every 8 hours orally	25 mg/kg every 12 hours orally	25 mg/kg every 8 hours orally	25 mg/kg every 8 hours orally	25 mg/kg every 6 hours orally
Penicillin G** aqueous	25,000 units/kg every 12 hours IM	25,000 units/kg every 12 hours IM	25,000 units/kg every 12 hours IM	25,000 units/kg every 12 hours IM	25,000 units/kg every 12 hours IM	25,000 units/kg every 12 hours IM
Amoxicillin/clavulanate^§^	25 mg amoxicillin/kg every 12 hours orally	25 mg amoxicillin/kg every 8 hours orally	25 mg amoxicillin/kg every 8 hours orally	25 mg amoxicillin/kg every 8 hours orally	25 mg amoxicillin/kg every 8 hours orally	25 mg amoxicillin/kg every 8 hours orally
Ciprofloxacin**	7.5 mg/kg every 12 hours orally	12.5 mg/kg every 12 hours orally	12.5 mg/kg every 12 hours orally	12.5 mg/kg every 12 hours orally	12.5 mg/kg every 12 hours orally	12.5 mg/kg every 12 hours orally
Clindamycin^§^	7 mg/kg every 8 hours orally	7 mg/kg every 8 hours orally	7 mg/kg every 8 hours orally	7 mg/kg every 8 hours orally	9 mg/kg every 8 hours orally	9 mg/kg every 8 hours orally
Doxycycline**^,††, §§^	5 mg/kg every 12 hours orally	5 mg/kg every 12 hours orally	5 mg/kg every 12 hours orally	5 mg/kg every 12 hours orally	5 mg/kg x 1dose orally, then 2.5 mg/kg every 12 hours orally	5 mg/kg x 1 dose orally, then 2.5 mg/kg every 12 hours orally
Levofloxacin**	10 mg/kg every 12 hours orally	10 mg/kg every 8 hours orally	10 mg/kg every 12 hours orally	10 mg/kg every 8 hours orally	10 mg/kg every 12 hours orally	10 mg/kg every 8 hours orally
**Alternative antimicrobial drug** ^¶¶^						
Moxifloxacin^††,^***	10 mg/kg every 24 hours orally	10 mg/kg every 24 hours orally	10 mg/kg every 24 hours orally	10 mg/kg every 24 hours orally	10 mg/kg every 24 hours orally	10 mg/kg every 24 hours orally
Linezolid^§,†††^	10 mg/kg every 12 hours orally	10 mg/kg every 8 hours orally	10 mg/kg every 12 hours orally	10 mg/kg every 8 hours orally	10 mg/kg every 12 hours orally	10 mg/kg every 8 hours orally
**Antitoxin (only to be used if antimicrobial drugs are not available or not appropriate; listed antitoxins joined by “or” are considered equivalent)**						
Raxibacumab^§§§^	55 mg/kg as a single dose IV	55 mg/kg as a single dose IV	55 mg/kg as a single dose IV	55 mg/kg as a single dose IV	55 mg/kg as a single dose IV	55 mg/kg as a single dose IV
or						
Obiltoxaximab^§§§^	16 mg/kg as a single dose IV	16 mg/kg as a single dose IV	16 mg/kg as a single dose IV	16 mg/kg as a single dose IV	16 mg/kg as a single dose IV	16 mg/kg as a single dose IV

**Abbreviations:** FDA = Food and Drug Administration; IM = intramuscular; IV = intravenous; PCN-S = penicillin-susceptible strains; PEP = postexposure prophylaxis; PEPAbx = antimicrobial postexposure prophylaxis for anthrax.

* Definitive therapy should be directed by antibiotic susceptibility test results, when available.

^†^ Up to 10% of naturally occurring strains of anthrax are penicillin- and amoxicillin-resistant; bioterror strains might be engineered to generate resistance to multiple antibiotics. Susceptibility results reported from CDC within 48–72 hours of initial isolation of anthrax.

^§^ Not approved by FDA for anthrax PEPAbx or treatment.

^¶^ Ampicillin can be used as an alternative to amoxicillin, if available.

** Approved by FDA for anthrax PEPAbx, treatment, or both, but specific uses (e.g., doses, dosing schedules, and patient populations) recommended in this report might differ from the FDA-approved labeling.

^††^ Instructions are available for preparing oral suspensions of moxifloxacin (**Source:** Hutchinson DJ, Johnson CE, Klein KC. Stability of extemporaneously prepared moxifloxacin oral suspensions. Am J Health Syst Pharm 2009;66:665–7.121) and doxycycline (**Source:** CDC. In an anthrax emergency: how to prepare doxycycline hyclate for children and adults who cannot swallow pills. Atlanta, GA: US Department of Health and Human Services, CDC; 2020. https://www.cdc.gov/anthrax/public-health/doxy-crushing-instruction-pamphlet.html).

^§§^ The use of tetracycline-class antibiotics during tooth development (i.e., last half of pregnancy, infancy, and childhood up to age 8 years) might cause permanent discoloration of the teeth (yellow, gray, or brown) and enamel hypoplasia. This adverse effect appears to occur less often with doxycycline but might occur more with longer durations of therapy.

^¶¶^ Alternative selections are for patients who have contraindications to or cannot tolerate first-line antimicrobial drugs or if first-line antimicrobial drugs are not available.

*** Not approved by FDA for any pediatric indications. Not approved by FDA for anthrax PEPAbx or treatment.

^†††^ Linezolid can be considered for PEP in scenarios when patients can receive regular monitoring for myelosuppression or neurotoxicity, which might occur within 14–28 days of use. If possible, switch to a different drug when available.

^§§§^ Premedicate with IV or oral diphenhydramine within 1 hour before administration. Hypersensitivity and anaphylaxis have been reported after raxibacumab and obiltoxaximab administration.

**TABLE 16:** Empiric* treatment regimens for preterm and full-term neonates 32–44 weeks’ postmenstrual age (gestational age plus chronologic age) with cutaneous anthrax without signs and symptoms of meningitis, by descending order of preference — CDC recommendations, 2023

Treatment	32 to <34 weeks’ gestational age		34 to <37 weeks’ gestational age		Full-term infant	
	0 to <1 week	1–4 weeks	0 to <1 week	1–4 weeks	0 to <1 week	1–4 weeks
	Dosage	Dosage	Dosage	Dosage	Dosage	Dosage
**First-line antimicrobial drug**						
Ciprofloxacin^†^	10 mg/kg every 12 hours orally	10 mg/kg every 12 hours orally	10 mg/kg every 12 hours orally	10 mg/kg every 12 hours orally	15 mg/kg every 12 hours orally	15 mg/kg every 12 hours orally
PCN-S only^§^:						
Amoxicillin^¶,^**	25 mg/kg every 12 hours orally	25 mg/kg every 8 hours orally	25 mg/kg every 8 hours orally	25 mg/kg every 8 hours orally	25 mg/kg every 8 hours orally	25 mg/kg every 8 hours orally
Penicillin VK^¶^	25 mg/kg every 12 hours orally	25 mg/kg every 8 hours orally	25 mg/kg every 12 hours orally	25 mg/kg every 8 hours orally	25 mg/kg every 8 hours orally	25 mg/kg every 6 hours orally
Amoxicillin/clavulanate^¶^	25 mg amoxicillin/kg every 12 hours orally	25 mg amoxicillin/kg every 8 hours orally	25 mg amoxicillin/kg every 12 hours orally	25 mg amoxicillin/kg every 8 hours orally	25 mg amoxicillin/kg every 8 hours orally	25 mg amoxicillin/kg every 8 hours orally
Doxycycline^†,††^	5 mg/kg every 12 hours orally	5 mg/kg every 12 hours orally	5 mg/kg every 12 hours orally	5 mg/kg every 12 hours orally	5 mg/kg x 1 dose orally, then 2.5 mg/kg every 12 hours orally	5 mg/kg x 1 dose orally, then 2.5 mg/kg every 12 hours orally
Clindamycin^¶^	7 mg/kg every 8 hours orally	7 mg/kg every 8 hours orally	7 mg/kg every 8 hours orally	7 mg/kg every 8 hours orally	9 mg/kg every 8 hours orally	9 mg/kg every 8 hours orally
Levofloxacin^†^	10 mg/kg every 12 hours orally	10 mg/kg every 8 hours orally	10 mg/kg every 12 hours orally	10 mg/kg every 8 hours orally	10 mg/kg every 12 hours orally	10 mg/kg every 8 hours orally
**Alternative antimicrobial drug^§§^**						
Moxifloxacin^††,¶¶^	10 mg/kg every 24 hours orally	10 mg/kg every 24 hours orally	10 mg/kg every 24 hours orally	10 mg/kg every 24 hours orally	10 mg/kg every 24 hours orally	10 mg/kg every 24 hours orally
Linezolid^¶^	10 mg/kg every 12 hours IV	10 mg/kg every 8 hours IV	10 mg/kg every 12 hours IV	10 mg/kg every 8 hours IV	10 mg/kg every 12 hours IV	10 mg/kg every 8 hours IV
Meropenem^¶^	13.3 mg/kg every 8 hours IV	20 mg/kg every 8 hours IV	20 mg/kg every 8 hours IV	20 mg/kg every 8 hours IV	20 mg/kg every 8 hours IV	20 mg/kg every 8 hours IV
Vancomycin^¶,^***	20 mg/kg loading dose IV, then 15 mg/kg every 12 hours IV	20 mg/kg loading dose IV, then 15 mg/kg every 12 hours IV	20 mg/kg loading dose IV, then 15 mg/kg every 8 hours IV	20 mg/kg loading dose IV, then 15 mg/kg every 8 hours IV	20 mg/kg loading dose IV, then 15 mg/kg every 8 hours IV	20 mg/kg loading dose IV, then 15 mg/kg every 8 hours IV
	Administer over a period of 1–2 hours. After dose 3 of vancomycin, adjust dosages to target AUC_24_ of 400 *µ*g x h/mL [preferred]; if AUC_24_ is not available, maintain trough concentrations of 10–15 *µ*g/mL. Check concentrations earlier if renal function is impaired. During the first 7–10 days, serum creatinine represents maternal concentration.					
Omadacycline^¶¶,†††^	NA	NA	NA	5.5 mg/kg loading dose IV x 1, then 3.85 mg/kg every 24 hours IV	5.5 mg/kg loading dose IV x 1, then 3.85 mg/kg every 24 hours IV	5.5 mg/kg loading dose IV x 1, then 3.85 mg/kg every 24 hours IV
Dalbavancin^¶^	NA	NA	NA	NA	NA	22.5 mg/kg x 1 dose IV
**Antitoxin (only to be used if antimicrobial drugs are not available or not appropriate; listed antitoxins joined by “or” are considered equivalent)**						
Raxibacumab^§§§^	55 mg/kg as a single dose IV	55 mg/kg as a single dose IV	55 mg/kg as a single dose IV	55 mg/kg as a single dose IV	55 mg/kg as a single dose IV	55 mg/kg as a single dose IV
or						
Obiltoxaximab^§§§^	16 mg/kg as a single dose IV	16 mg/kg as a single dose IV	16 mg/kg as a single dose IV	16 mg/kg as a single dose IV	16 mg/kg as a single dose IV	16 mg/kg as a single dose IV
AIGIV	1 vial (approximately 60 units) as a single dose IV	1 vial (approximately 60 units) as a single dose IV	1 vial (approximately 60 units) as a single dose IV	1 vial (approximately 60 units) as a single dose IV	1 vial (approximately 60 units) as a single dose IV	1 vial (approximately 60 units) as a single dose IV

**Source:** Bradley JS, Nelson JD, eds. Nelson’s pediatric antimicrobial therapy. 29th ed. Itasca, IL: American Academy of Pediatrics; 2023.

**Abbreviations:** FDA = Food and Drug Administration; IV = intravenous; NA = not applicable; PCN-S = penicillin-susceptible strains; PEPAbx = antimicrobial postexposure prophylaxis for anthrax.

* Definitive therapy should be directed by antibiotic susceptibility test results, when available.

^†^ Approved by FDA for anthrax PEPAbx, treatment, or both, but specific uses (e.g., doses, dosing schedules, and patient populations) recommended in this report might differ from the FDA-approved labeling.

^§^ Up to 10% of naturally occurring strains of anthrax are resistant to penicillin and amoxicillin; bioterror strains might be engineered to generate resistance to multiple antibiotics. Susceptibility results reported from CDC within 48–72 hours of initial isolation of anthrax.

^¶^ Not approved by FDA for anthrax PEPAbx or treatment.

** Ampicillin can be used as an alternative to amoxicillin, if available.

^††^ Instructions are available for preparing oral suspensions of moxifloxacin (**Source:** Hutchinson DJ, Johnson CE, Klein KC. Stability of extemporaneously prepared moxifloxacin oral suspensions. Am J Health Syst Pharm 2009;66:665–7.121) and doxycycline (**Source:** CDC. In an anthrax emergency: how to prepare doxycycline hyclate for children and adults who cannot swallow pills. Atlanta, GA: US Department of Health and Human Services, CDC; 2020. https://www.cdc.gov/anthrax/public-health/doxy-crushing-instruction-pamphlet.html).

^§§^ Alternative selections are for patients who have contraindications to or cannot tolerate first-line antimicrobial drugs or if first-line antimicrobial drugs are not available.

^¶¶^ Not approved by FDA for any pediatric indications. Not approved by FDA for anthrax PEPAbx or treatment.

*** Allergic reactions are rare in neonates, but neonates can release histamine that causes hypotension after rapid infusions of vancomycin; thus, it can be safest to pretreat with an antihistamine.

^†††^ The use of tetracycline-class antibiotics during tooth development (i.e., last half of pregnancy, infancy, and childhood up to age 8 years) might cause permanent discoloration of the teeth (yellow, gray, or brown) and enamel hypoplasia. This adverse effect appears to occur less often with doxycycline but might occur more with longer durations of therapy.

^§§§^ Premedicate with IV or oral diphenhydramine within 1 hour before administration. Hypersensitivity and anaphylaxis have been reported after raxibacumab and obiltoxaximab administration.

**TABLE 17:** Empiric* treatment regimens for preterm and full-term neonates 32–44 weeks’ postmenstrual age (gestational age plus chronologic age) with systemic^†^ anthrax with or without meningitis, by descending order of preference — CDC recommendations, 2023

Regimen				Example			
Regimen 1. Two bactericidal drugs from different antimicrobial drug classes plus a PSI or an RNAI				Ciprofloxacin plus meropenem plus clindamycin^§^			
Regimen 2. One bactericidal drug plus a PSI				Meropenem plus doxycycline			
Regimen 3. One bactericidal drug plus a second bactericidal drug from a different antimicrobial drug class				Meropenem plus ciprofloxacin			
Regimen 4. One bactericidal drug plus an RNAI (rifampin should not be used as monotherapy)				Meropenem plus rifampin			
Regimen 5. A PSI plus an RNAI (rifampin should not be used as monotherapy)				Doxycycline plus rifampin			
Regimen 6. Two PSIs from different antimicrobial drug classes				Linezolid plus doxycycline			
Regimen 7. A single bactericidal drug				Meropenem			
Regimen 8. A single PSI				Doxycycline or clindamycin			
**Treatment**	**Mechanism of action**	**32 to <34 weeks’ gestational age**		**34 to <37 weeks’ gestational age**		**Full-term infant**	
		**0 to <1 week**	**1–4 weeks**	**0 to <1 week**	**1–4 weeks**	**0 to <1 week**	**1–4 weeks**
		**Dosage**	**Dosage**	**Dosage**	**Dosage**	**Dosage**	**Dosage**
**First-line antimicrobial drug^¶^**							
Ciprofloxacin**	C	7.5 mg/kg every 12 hours IV	12.5 mg/kg every 12 hours IV	12.5 mg/kg every 12 hours IV	12.5 mg/kg every 12 hours IV	12.5 mg/kg every 12 hours IV	12.5 mg/kg every 12 hours IV
Levofloxacin**	C	10 mg/kg every 12 hours IV	10 mg/kg every 8 hours IV	10 mg/kg every 12 hours IV	10 mg/kg every 8 hours IV	10 mg/kg every 12 hours IV	10 mg/kg every 8 hours IV
Meropenem^††^	C	13.3 mg/kg every 8 hours IV	20 mg/kg every 8 hours IV	20 mg/kg every 8 hours IV	20 mg/kg every 8 hours IV	20 mg/kg every 8 hours IV	20 mg/kg every 8 hours IV
PNC-S only^§§^:							
Penicillin G**^,¶¶^ aqueous	C	100,000 units/kg every 12 hours IV/IM	100,000 units/kg every 8 hours IV/IM	100,000 units/kg every 8 hours IV/IM	100,000 units/kg every 6 hours IV/IM	100,000 units/kg every 8 hours IV/IM	100,000 units/kg every 6 hours IV/IM
Ampicillin^††^	C	50 mg/kg every 12 hours IV/IM	75 mg/kg every 12 hours IV/IM	50 mg/kg every 8 hours IV/IM	50 mg/kg every 8 hours IV/IM	50 mg/kg every 8 hours IV/IM	50 mg/kg every 8 hours IV/IM
Vancomycin^††,^***	C	20 mg/kg loading dose IV, then 15 mg/kg every 12 hours IV	20 mg/kg loading dose IV, then 15 mg/kg every 12 hours IV	20 mg/kg loading dose IV, then 15 mg/kg every 8 hours IV	20 mg/kg loading dose IV, then 15 mg/kg every 8 hours IV	20 mg/kg loading dose IV, then 15 mg/kg every 8 hours IV	20 mg/kg loading dose IV, then 15 mg/kg every 8 hours IV
		Administer over a period of 1–2 hours. After dose 3 of vancomycin, adjust dosages to target AUC_24_ of 400 *µ*g x h/mL [preferred]; if AUC_24_ is not available, maintain trough concentrations of 10–15 *µ*g/mL. Check concentrations earlier if renal function is impaired. During the first 7–10 days, serum creatinine represents maternal concentration.					
Doxycycline**	PSI	5 mg/kg every 12 hours IV	5 mg/kg every 12 hours IV	5 mg/kg every 12 hours IV	5 mg/kg every 12 hours IV	5 mg/kg x 1 dose IV, then 2.5 mg/kg every 12 hours	5 mg/kg x 1 dose IV, then 2.5 mg/kg every 12 hours
**Alternative antimicrobial drug^†††^**							
Moxifloxacin^§§§^	C	10 mg/kg every 24 hours IV	10 mg/kg every 24 hours IV	10 mg/kg every 24 hours IV	10 mg/kg every 24 hours IV	10 mg/kg every 24 hours IV	10 mg/kg every 24 hours IV
Imipenem^††,¶¶¶^	C	25 mg/kg every 8 hours IV infused over 1.5 hours	25 mg/kg every 8 hours IV infused over 1.5 hours	25 mg/kg every 8 hours IV infused over 1.5 hours	25 mg/kg every 8 hours IV infused over 1.5 hours	25 mg/kg every 8 hours IV infused over 1.5 hours	25 mg/kg every 8 hours IV infused over 1.5 hours
Dalbavancin^††^	C	NA	NA	NA	NA	NA	22.5 mg/kg x 1 dose IV
PCN-S only^††^:							
Ampicillin/sulbactam^††^	C	50 mg ampicillin/kg every 12 hours IV	50 mg ampicillin/kg every 12 hours IV	50 mg ampicillin/kg every 12 hours IV	50 mg ampicillin/kg every 12 hours IV	50 mg ampicillin/kg every 12 hours IV	50 mg ampicillin/kg every 12 hours IV
Clindamycin^††^	PSI	7 mg/kg every 8 hours IV/IM	7 mg/kg every 8 hours IV/IM	7 mg/kg every 8 hours IV/IM	7 mg/kg every 8 hours IV/IM	9 mg/kg every 8 hours IV/IM	9 mg/kg every 8 hours IV/IM
Linezolid^††^	PSI	10 mg/kg every 12 hours IV	10 mg/kg every 8 hours IV	10 mg/kg every 12 hours IV	10 mg/kg every 8 hours IV	10 mg/kg every 12 hours IV	10 mg/kg every 8 hours IV
Rifampin^††,^****	RNAI	10 mg/kg every 24 hours IV	10 mg/kg every 24 hours IV	10 mg/kg every 24 hours IV	10 mg/kg every 24 hours IV	10 mg/kg every 24 hours IV	10 mg/kg every 24 hours IV
Omadacycline^§§§,††††^	PSI	NA	NA	NA	5.5 mg/kg loading dose IV x 1, then 3.85 mg/kg every 24 hours IV	5.5 mg/kg loading dose IV x 1, then 3.85 mg/kg every 12 hours IV	5.5 mg/kg loading dose IV x 1, then 3.85 mg/kg every 12 hours IV
**Antitoxin (only to be used if antimicrobial drugs are not available or not appropriate; listed antitoxins joined by “or” are considered equivalent)**							
Raxibacumab^§§§§^		55 mg/kg as a single dose IV	55 mg/kg as a single dose IV	55 mg/kg as a single dose IV	55 mg/kg as a single dose IV	55 mg/kg as a single dose IV	55 mg/kg as a single dose IV
or							
Obiltoxaximab^§§§§^		32 mg/kg as a single dose IV	32 mg/kg as a single dose IV	32 mg/kg as a single dose IV	32 mg/kg as a single dose IV	32 mg/kg as a single dose IV	32 mg/kg as a single dose IV
AIGIV		1 vial (approximately 60 units) as a single dose IV	1 vial (approximately 60 units) as a single dose IV	1 vial (approximately 60 units) as a single dose IV	1 vial (approximately 60 units) as a single dose IV	1 vial (approximately 60 units) as a single dose IV	1 vial (approximately 60 units) as a single dose IV

**Abbreviations:** AIGIV = anthrax immunoglobulin intravenous; C = bactericidal; FDA = Food and Drug Administration; IM = intramuscular; IV = intravenous; NA = not applicable; PCN-S = penicillin-susceptible strains; PEPAbx = antimicrobial postexposure prophylaxis for anthrax; PSI = protein synthesis inhibitor; RNAI = RNA synthesis inhibitor.

* Definitive therapy should be directed by antibiotic susceptibility test results, when available.

^†^ “Systemic” was defined as one or more of the following using cutoffs for adults aged ≥18 years: hyperthermia or hypothermia, tachycardia, tachypnea, hypotension, or neutrophilia or neutropenia. (**Source:** Katharios-Lanwermeyer S, Holty JE, Person M, et al. Identifying meningitis during an anthrax mass casualty incident: systematic review of systemic anthrax since 1880. Clin Infect Dis 2016;62:1537–45).

^§^ For anthrax meningitis, consider using antimicrobial drugs that have demonstrated potential neuroprotective benefits in vivo (e.g., doxycycline, clindamycin, and ß-lactam antimicrobial drugs).

^¶^ For highly bioavailable antimicrobial drugs (e.g., ciprofloxacin, doxycycline, and linezolid), if the IV formulation is not available, oral formulations can be considered for patients with an intact gastrointestinal tract where absorption is expected to be complete after oral administration.

** Approved by FDA for anthrax PEPAbx, treatment, or both, but specific uses (e.g., doses, dosing schedules, and patient populations) recommended in this report might differ from the FDA-approved labeling.

^††^ Not approved by FDA for anthrax PEPAbx or treatment.

^§§^ Up to 10% of naturally occurring strains of anthrax are resistant to penicillin and amoxicillin; bioterror strains might be engineered to generate resistance to multiple antibiotics. Susceptibility results reported from CDC within 48–72 hours of initial isolation of anthrax.

^¶¶^ Penicillin G benzathine or procaine should never be administered intravenously.

*** Vancomycin does not cross an intact blood-brain barrier but might cross with meningitis because of breakdown of the barrier. Allergic reactions are rare in neonates; however, neonates can release histamine that causes hypotension after rapid infusions of vancomycin, thus it might be safest to pretreat with an antihistamine.

^†††^ Alternative selections are for patients who have contraindications to or cannot tolerate first-line antimicrobial drugs or if first-line antimicrobial drugs are not available.

^§§§^ Not approved by FDA for any pediatric indications. Not approved by FDA for anthrax PEPAbx or treatment.

^¶¶¶^ If meningitis is confirmed, imipenem moves to a first-line antimicrobial drug.

**** Rifampin is an RNAI and also bactericidal; however, it should not be used as monotherapy.

^††††^ The use of tetracycline-class antibiotics during tooth development (i.e., last half of pregnancy, infancy, and childhood up to age 8 years) might cause permanent discoloration of the teeth (yellow, gray, or brown) and enamel hypoplasia. This adverse effect appears to occur less often with doxycycline but might occur more with longer durations of therapy.

^§§§§^ Premedicate with IV or oral diphenhydramine within 1 hour before administration. Hypersensitivity and anaphylaxis have been reported after raxibacumab and obiltoxaximab administration.

##### PEP and Treatment Regimens for Cutaneous Anthrax Without Signs and Symptoms of Meningitis

For preterm and full-term newborns 32–44 weeks’ postmenstrual age (i.e., gestational age plus chronologic age), empiric PEP (Table 15), and empiric cutaneous anthrax treatment (Table 16) regimens include either a single antimicrobial drug or a single antitoxin. These regimens are summarized as follows:

- Antimicrobial drug: Choose a single antimicrobial drug.
  ° Antimicrobial drugs are listed in descending order of preference in the table. Listed drugs joined by “or” are considered equivalent.
  ° Continue or switch antimicrobial drug based on susceptibility testing once available.
  ° Only choose a “PCN-S only” antimicrobial drug after the strain has been determined to be penicillin susceptible.
- Antitoxin: Choose a single antitoxin if no antimicrobial drugs are available.

For preterm and full-term newborns 32–44 weeks’ postmenstrual age (i.e., gestational age plus chronologic age), PEPAbx after aerosol exposure should continue for 60 days (*33*). Vaccine is not currently indicated for this age group. PEPAbx after nonaerosol exposure should continue for 7 days.

For preterm and full-term newborns with cutaneous anthrax without signs and symptoms of meningitis, the treatment regimen should continue for 7–10 days, or until clinical criteria for stability are met. If an aerosol exposure might have occurred, patients should transition from a treatment to a PEP regimen (Table 15); the combined regimen should total 60 days from exposure. If no aerosolizing event occurred, patients with cutaneous anthrax do not need to continue PEPAbx.

##### Treatment Regimens for Systemic Anthrax With or Without Meningitis

For preterm and full-term newborns 32–44 weeks’ postmenstrual age (i.e., gestational age plus chronologic age), empiric treatment regimens for those with systemic anthrax with or without meningitis (Table 17) are summarized as follows:

- Antimicrobial drugs: Choose two bactericidal drugs from different antimicrobial drug classes plus a PSI or an RNAI.
  º Antimicrobial drugs are listed in descending order of preference in the table. Listed drugs joined by “or” are considered equivalent.
  º Continue or switch antimicrobial drugs based on susceptibility testing once available.
  º Only choose a “PCN-S only” antimicrobial drug after the strain has been determined to be penicillin susceptible.
- Antitoxin: Choose a single antitoxin as adjunctive therapy.

If an appropriate combination of bactericidal drugs plus a PSI or an RNAI is contraindicated, not well tolerated, or not available or if meningitis is considered unlikely, consider the following regimens in descending order of preference:

- One bactericidal drug plus a PSI (start with this regimen if meningitis is not suspected)
- One bactericidal drug plus a second bactericidal agent from a different antimicrobial drug class
- One bactericidal drug plus an RNAI
- A PSI plus an RNAI
- Two PSIs from different antimicrobial drug classes
- A single bactericidal drug
- A single PSI

From a PK/PD perspective, minocycline and doxycycline are the preferred PSIs because they provide more robust drug exposures in plasma and CSF compared with macrolides or clindamycin. A single RNAI (i.e., rifampin) should not be used as monotherapy because of the potential for rapid development of resistance (*135*). In addition, when meningitis is not suspected, certain oral formulations are included as alternatives in case IV formulations are not available.

Duration of antimicrobial drug treatment should be for 2 weeks or longer, although as immune-compromised hosts, neonates might require a longer duration of therapy to achieve cure. Transition from IV administration to oral medication for neonates tolerating regular feeding should be based on patient improvement and clinical judgment. Patients with naturally acquired noninhalation cases do not need continuation of antimicrobial drug therapy for PEP. If an aerosol exposure might have occurred (i.e., a bioterrorism-related incident or animal skin drum–related event), preterm and full-term newborns (who are not considered fully immunocompetent) should transition to an oral PEP regimen (Table 15). The total duration of antimicrobial drug therapy (i.e., treatment plus PEP) should be 60 days from onset of illness.

### Special Considerations for Inhalation and Ingestion Anthrax

Pleural effusion and other fluid collections are common complications of anthrax (*28*,*36*). Hypothetically, draining pleural fluid or ascites might reduce the amount of lethal factor, thereby reducing illness severity and decreasing mortality. In addition, drainage of pleural fluid is believed to improve survival by decreasing mechanical lung compression. Early and aggressive drainage of any clinically or radiographically apparent pleural effusion is recommended; chest tube drainage is preferred over thoracentesis because many effusions will require prolonged drainage. Thoracotomy or video-assisted thoracic surgery might be required to remove gelatinous or loculated collections. Ascites should also be drained, if feasible, and monitored for reaccumulation; continuous drainage might be required. Standard precautions are sufficient when caring for anthrax patients. The exception is when the patient is potentially contaminated with *B. anthracis* spores. In such cases, the patient should be isolated in an airborne infection isolation room until decontamination is completed (*172*).

### Special Considerations for Anthrax Meningitis

#### Diagnosis

 Anthrax meningitis has a mortality rate that approaches 100% (*29*,*38*) and is a common complication of anthrax. Meningitis can either be primary (i.e., have no obvious route of transmission) or secondary (i.e., develop as a complication of any other form of anthrax). Depending on the route of transmission, 14%–37% of patients with injection, ingestion, systemic cutaneous, or inhalation anthrax develop meningitis (*38*). Thus, all patients with symptoms or signs of systemic disease should be evaluated for meningitis. In a wide-area aerosol release of *B. anthracis* spores mass casualty event, conventional standards of care for diagnosing meningitis (i.e., imaging and lumbar puncture followed by analysis of CSF) might be limited or not available. For such situations, a screening tool has been developed to identify patients likely to have anthrax meningitis. On the basis of this screening tool (Figure), patients are likely to have meningitis if they have either

- ≥2 of the following signs or symptoms: severe headache, altered mental status, meningeal signs, or other neurologic deficits, or
- ≥1 of the following signs or symptoms: severe headache, altered mental status, meningeal signs, or other neurologic deficits and ≥1 of the following signs or symptoms: nausea/vomiting, abdominal pain, or fever (either subjective or measured) or chills.

Patients are unlikely to have meningitis if they do not have severe headache, altered mental status, meningeal signs, and other neurologic deficits. Patients who have bacteremia; those with obesity, diabetes, hypertension, and chronic obstructive pulmonary disease; and current and former smokers appear to be at increased risk for meningitis.

#### Adjunctive Therapy

 The combined effects of infection and intercranial bleeding predispose patients to malignant, rapidly fatal brain swelling and elevated intracranial pressure. Mannitol or hypertonic saline should be considered for patients with anthrax meningitis and evidence of cerebral edema (*98*). The data did not demonstrate a survival benefit in those who received steroids compared with those who did not. However, steroids did not appear to cause harm and should be used if clinically indicated. In addition, therapies that target intracranial bleeding and swelling (e.g., nimodipine) have been reported to improve outcomes in aneurysmal subarachnoid hemorrhage and intracerebral hemorrhage and might be applicable to the treatment of hemorrhagic anthrax meningitis. However, at present no data from animal studies or human patients with anthrax are available to support the theoretical benefit of these treatments (*51*).

## Research Needs

Development of a wide array of medical countermeasures that can lessen the morbidity and mortality of anthrax, especially from bioengineered *B. anthracis* strains*,* will make anthrax a less desirable bioweapon. To attain preparedness goals, additional diverse countermeasures are needed to address multidrug-resistant *B. anthracis*. Newer antimicrobial drugs with novel mechanisms might largely mitigate certain challenges presented by multidrug-resistant strains. However, PK/PD data, on which rational dosing would be based, are limited for these newer antimicrobial drugs. More research is needed on CSF penetration and PK/PD topics (including PK/PD targets for *B. anthracis*) to enhance current and newly developed medical countermeasures. These research gaps are especially profound for children and pregnant and lactating persons. In addition, animal and in vitro studies are needed to assess potential synergism (or antagonism) of antimicrobial drug combinations and antitoxin dosing and the benefit of inhibiting toxin production by a PSI.

Considerable gaps also exist in knowledge of how ß-lactam antimicrobial drugs bind to multiple target receptors in *B. anthracis*. Acquisition of receptor binding data would support optimizing ß-lactam combination therapies with and without addition of established or newer ß-lactamase inhibitors. The utility of current antitoxin countermeasures is considerably compromised when anthrax toxins become intracellular. To overcome this gap, studies are needed to develop small-molecule inhibitors. Such agents might be of use in patients during the fulminant phase of disease. Studies are needed on the immunogenicity and safety of currently approved anthrax vaccines in special populations, including pregnant and lactating persons, children, and older adults. Vaccines that enhance immunogenicity sooner also would be desirable.

Most deaths after a wide-area aerosol release of *B. anthracis* spores would likely be from complications of anthrax meningitis (e.g., intracranial bleeding). Unlike other bacterial meningitides, anthrax meningitis is characterized by substantial bleeding and cerebral edema. Previously, neither clinical guidelines nor basic research have focused on the bleeding or cerebral edema that accompanies meningitis or on drugs or molecules that might be neuroprotective for this complication. The best available PK/PD recommendations are provided by predicting antimicrobial drug exposures in plasma and CSF via Monte Carlo simulations. Whereas these analyses were helpful to rank antimicrobial drugs, the inherent limitations of borrowing PK/PD exposure targets from pathogens other than *B. anthracis* require the PK/PD results to be interpreted conservatively and considered in conjunction with animal efficacy and clinical data. Finally, little is known about host factors that might predispose a person to develop anthrax meningitis.

## Conclusion

Anthrax continues to occur in certain places around the world, with an estimated 20,000–100,000 cases occurring annually (*173*). *B. anthracis* also continues to be considered the most likely bioweapon to be used during a bioterrorist event because of its availability, ease of dissemination, and high mortality rate associated with systemic anthrax. Biopreparedness efforts are made more challenging by the ease with which *B. anthracis* can be made resistant to first-line antimicrobial drugs for PEP and treatment. This report describes updated CDC guidelines and recommendations for the preferred prevention and treatment regimens for naturally occurring anthrax. Also provided are a wide range of alternative regimens to first-line antimicrobial drugs for use if patients have contraindications or intolerances or after a wide-area aerosol release of *B. anthracis* spores if resources become limited or a multidrug-resistant *B. anthracis* strain is used. Future revisions to these guidelines will be supported by new research and technological advancements for prevention and clinical management of anthrax.
