# *Erratum:* Vol. 72, No. RR-6

**DOI:** 10.15585/mmwr.mm7331a4

**Published:** 2024-08-08

In the Recommendation and Report “CDC Guidelines for the Prevention and Treatment of Anthrax, 2023,” multiple errors occurred. 

On page 32, in Table 7, and page 35, in Table 10, the imipenem/cilastatin dose should have read **1 **g every 6 hours.

On page 33, in the Abbreviations under Table 8, the abbreviation for PCN-S should have read penicillin-**susceptible** strains.

On page 37, in Table 12, the clarithromycin dose should have read **7.5** mg/kg.

On page 37, in Table 12, and page 38, in Table 13, the age range for the last two doses of moxifloxacin listed should have read **≥12** to <18 years.

On page 39, in Table 13, and page 41, in Table 14, the first dose of AIGIV listed should have read **<10** kg.

On page 42, in Table 15; page 43, in Table 16; and page 45, in Table 17, the moxifloxacin dose in all columns should have read **10** mg/kg.

On page 42, in Table 15, the amoxicillin dose should have read **25** mg/kg in all columns, and the linezolid dose in the 0 to <1 week columns should have read every **12** hours.

On page 45, in Table 17, the omadacycline dose in the last three columns should have read 5.5 mg/kg loading dose **IV **× 1, then 3.85 mg/kg **every** **24 hours IV**.

